# RecA-like domain 2 of DNA-dependent ATPase A domain, a SWI2/SNF2 protein, mediates conformational integrity and ATP hydrolysis

**DOI:** 10.1042/BSR20180568

**Published:** 2018-06-27

**Authors:** Ritu Bansal, Vijendra Arya, Ramesh Sethy, Radhakrishnan Rakesh, Rohini Muthuswami

**Affiliations:** School of Life Sciences, Jawaharlal Nehru University, New Delhi 110067, India

**Keywords:** Chromatin remodeling, Helicases, SWI2/SNF2 proteins, SMARCAL1

## Abstract

ATP-dependent chromatin remodeling proteins use the energy released from ATP hydrolysis to reposition nucleosomes in DNA-dependent processes. These proteins are classified as SF2 helicases. SMARCAL1, a member of this protein family, is known to modulate both DNA repair and transcription by specifically recognizing DNA molecules possessing double-strand to single-strand transition regions. Mutations in this gene cause a rare autosomal recessive disorder known as Schimke Immuno-Osseous Dysplasia (SIOD).

Structural studies have shown that the ATP-dependent chromatin remodeling proteins possess two RecA-like domains termed as RecA-like domain 1 and RecA-like domain 2. Using Active DNA-dependent ATPase A domain (ADAAD), the bovine homolog of SMARCAL1, as a model system we had previously shown that the RecA-like domain 1 containing helicase motifs Q, I, Ia, II, and III are sufficient for ligand binding; however, the Rec A-like domain 2 containing motifs IV, V, and VI are needed for ATP hydrolysis. In the present study, we have focused on the motifs present in the RecA-like domain 2. Our studies demonstrate that the presence of an aromatic residue in motif IV is needed for interaction with DNA in the presence of ATP. We also show that the motif V is required for the catalytic efficiency of the protein and motif VI is needed for interaction with DNA in the presence of ATP. Finally, we show that the SIOD-associated mutation, R820H, present in motif VI results in loss of ATPase activity, and therefore, reduced response to DNA damage.

## Introduction

The SWI2/SNF2 proteins, also known as ATP-dependent chromatin remodeling proteins, utilize the energy released from ATP hydrolysis to mediate myriad functions in the cell [1–5]. These proteins have been classified as helicases due to the presence of the conserved helicase motifs—Q, I, Ia, II, III, IV, V, and VI [6]. The crystal structure of Rad54 and Chd1, members of the protein family, has shown that these proteins, like other classical helicases, contain the two RecA-like domains—RecA-like domain 1 and RecA-like domain 2 [7–9]. Motifs Q, I, Ia, II, and III are present in RecA-like domain 1 while motifs IV, V, and VI are present in RecA-like domain 2. The two domains are separated by a linker region that can vary in length between the various members of the SW12/SNF2 proteins [10].

Comparison of the crystal structures of SWI2/SNF2 proteins with that of SF1 and SF2 helicases has shown both similarities and differences [11]. For example, motifs I and II are highly conserved between SWI2/SNF2 proteins and other helicases but motifs Ia and III show some structural differences between the two classes of proteins [9,11]. In case of RecA-like domain 2, motif IV is structurally conserved between the SWI2/SNF2 proteins and classical helicases but motifs V and VI show structural divergence [9].

The role of helicase motifs in ligand binding and ATP hydrolysis has been characterized in both SF1 and SF2 helicases [12]. Motif I is important for ATP hydrolysis and in many cases also mediates ATP binding [13,14]. Motif II, also known as Walker B box, is known to interact with magnesium ion. Motif III has been shown to function as a sensor for ATP hydrolysis [15,16]. Motif IV has been shown to interact with the phosphate backbone of the DNA [17,18]. Motif V has also been reported to interact with DNA while motif VI has been shown to interact with ATP in case of SF1 helicase [19] and with DNA/RNA in case of SF2 helicases [18,20–22].

In contrast, very less information is available about the role of these motifs in case of SWI2/SNF2 proteins. Mutational analysis of the Snf2 protein from *Saccharomyces cerevisiae* has shown conserved residues required for function in each of the motifs [23]. Studies with Active DNA-dependent ATPase A domain (ADAAD), the bovine homolog of SMARCAL1, had shown that the protein can bind to both DNA and ATP independently; however, binding of DNA induces a conformational change that allows ATP to bind with 10-fold higher affinity and similarly binding of ATP induces a conformational change that allows DNA to bind with higher affinity [24]. Mutational analysis showed that motifs Q and I are required for ATP hydrolysis but not for ATP binding [25].

Human SMARCAL1, an annealing helicase that has role both in DNA damage response as well as transcription regulation, has been linked to Schimke Immuno-Osseous Dysplasia (SIOD) and analysis of three mutations, which map to RecA-like domain 1, present in these patients showed that these mutants cannot hydrolyze ATP [26–30]. Further, binding studies showed that upon binding to ATP, the mutants do not undergo the requisite conformational change and thus cannot bind to DNA with higher affinity [30].

The role of the motifs present in the RecA-like domain 2 has not yet been characterized and therefore, the focus of this paper is to characterize the function of these motifs using ADAAD as the model system. We show that an aromatic residue in motif IV is needed for interaction with DNA. The motif V appears to a play a role in dictating the catalytic efficiency of the protein while motif VI is needed for interaction with DNA. Further, the major role of the motifs present in the RecA-like domain 2 appears to be in maintaining the conformational integrity but not the conformational stability of the protein. Finally, we have also characterized the SIOD-associated mutation, R820H, and show that this mutation leads to reduced DNA binding in the presence of ATP and therefore, loss in ATPase activity.

## Methods

### Chemicals

All chemicals used in the present study were purchased from either Merck or Sigma-Aldrich unless otherwise stated. The stem−loop DNA (slDNA: 5′-GCGCAATTGCGCTCGACGATTTTTTAGCGCAATTGCGC-3′) as well as the primers (Supplementary Table S1) used for making the site-directed mutants were synthesized by Sigma-Aldrich. PCR enzymes, restriction enzymes, and Dpn1 were purchased from New England Biolabs (U.S.A.) and Merck (India).

### Construction of site-directed mutants and protein purification

The plasmid, pCP101, containing the gene expressing ADAAD cloned between NdeI and XhoI sites of pET-14b, was used for creating site-directed mutations [24]. All the mutants described in the present study were generated by PCR amplification using specific primers and the mutations were confirmed by sequencing. *E. coli* BL21 (DE3) were transformed with these mutant plasmids for protein production. Protein expression was induced by 0.5 mM IPTG at 16°C for 16 h. The cells were harvested by centrifugation at 5000 rpm at 4°C and resuspended in lysis buffer containing 50 mM Tris-Cl (pH 8.0), 150 mM NaCl, 150 mM MgCl_2_, 0.1% (v/v) Triton X-114, 0.2 mg/ml lysozyme, 10 mM β-mercaptoethanol, and 0.5 mM PMSF. The cells were incubated at 4°C for 1 h and lysed by sonication (15 s ON and 45 s OFF; five cycles). After sonication, the cell debris was removed by centrifugation at 10000 rpm for 45 min at 4°C. The supernatant, thus, obtained was loaded onto a Ni^2+^-NTA column. The column was washed with wash buffer (50 mM Tris-Cl (pH 8.0), 150 mM NaCl, 150 mM MgCl_2_, 10 mM β-mercaptoethanol, 30 mM imidazole, and 0.5 mM PMSF) and the bound protein was eluted with buffer containing 250 mM imidazole, 50 mM Tris-Cl (pH 8.0), 5 mM MgCl_2_, 100 mM NaCl, and 5 mM β-mercaptoethanol. The eluted fractions were pooled together and dialyzed against dialysis buffer containing 20 mM Tris-SO_4_ (pH 7.5), 20% (v/v) glycerol, 1 mM EDTA, and 50 mM K_2_SO_4_ till the conductivity of the protein was same as that of the dialysis buffer. The dialyzed protein was loaded onto DEAE-sepharose ion-exchange column pre-equilibrated with dialysis buffer and purified fractions obtained were analyzed by SDS/PAGE. The concentration of the purified protein was determined using Bradford reagent. The purification of all the proteins reported in the present study is present in Supplementary Figure S1. Each protein was purified at least three times and both binding and CD studies were performed with the independently purified protein to verify the data.

### Fluorescence studies

Steady-state fluorescence titrations were assayed using Cary Eclipse fluorimeter. The protein was excited at 295 nm and the emission was measured at 340 nm. The binding data were fit to one-site saturation for the ligand and protein interaction. The following equation was used to calculate the dissociation constant (*K*_d_):

$$\Delta F/F\text{o}=B_{\max}[\text{L}]/(K_{\text{d}}+[\text{L}])$$

where *B*_max_ is the maximal binding, [L] is the ligand concentration, and *K*_d_ is the dissociation constant.

The spectra were corrected for dilutions and inner filter effect. Care was taken to ensure that the volume of ligand added was equal or less than 10% of the starting reaction. As studied earlier, the ATPase activity of protein with ligands is negligible at 25°C and in the absence of regeneration buffer [24]. Therefore, all the fluorescence studies showed the interaction between protein and ligands in the absence of ATP hydrolysis. Representative spectra are shown in Supplementary Figure S2.

In the experiments involving saturating concentrations of either DNA or ATP, 2 μM DNA and 20 µM ATP were used to saturate 0.5 μM protein.

### ATPase assay

NADH coupled oxidation assay was used to monitor the ATPase activity using 1.4 μg of protein in REG buffer containing 50 mM Tris-SO_4_ (pH 7.5), 1 mM MgSO_4_, 5 mM β-mercaptoethanol, 10 mM phosphoenolpyruvate, and 150 μg/ml pyruvate kinase along with 2 mM ATP, 0.1 mg/ml NADH, and 10 nM stem–loop DNA in a 250 μl of reaction volume. The reaction was incubated for 15 min at 37°C and the absorbance of NADH was measured at 340 nm using microplate spectrophotometer.

### CD spectroscopy

Far UV CD spectra were obtained by Chirascan (Applied Photophysics) using a 1 mm cuvette. The reaction contained 0.1 mg/ml protein, reaction buffer (20 mM Tris-Cl (pH 7.5), 1 mM EDTA, 100 mM NaCl, 1 mM MgCl_2_, and 1 mM DTT), 20 µM ATP, and 2 µM DNA. Thermal data for all the mutants were obtained by changing the temperature from 25 to 90°C and monitoring the CD curve. CD values are reported in terms of mean residue ellipticity (*θ*), and was calculated by the formula [*θ*] = (*S* × mRw)/(10*cl*), where *S* is the CD signal in millidegrees, mRw is the mean residue mass, *c* is the concentration of the protein in mg/ml, and *l* is the path length in cm.

In titration with ligands, 2 μM DNA and 20 µM ATP were used to saturate 0.1 mg/ml protein.

### Immunofluorescence

For RNase treatment, HeLa cells were grown on a coverslip in a 35 mm culture dish. After attaining 50% confluency, cells were treated with 2 μM doxorubicin for 10 min followed by the washing of cells with 1× PBS (four times). Subsequently, cells were permeablized with 0.5% TritonX-100 in 1× PBS for 20 min at 4°C. The cells were again washed with 1× PBS and treated with 1 mg/ml RNase for 20 min at room temperature. The cells were fixed with 1:1 methanol, acetone mixture for 10 min and permeabilized with 0.5% (v/v) Triton X-100. After permeabilization, the cells were washed with 1× PBS followed by blocking with 2% BSA at 37°C for 1 h. The cells were again washed with 1× PBS and incubated with required amount of primary antibody in 2% BSA at 37°C for 1 h. The cells were then washed with 1× PBS 3–4 times, incubated with a mixture of TRITC or FITC-conjugated secondary antibodies and DAPI/Hoechst 33342 at a dilution of 1:1000 in 2% BSA at 37°C for 1 h. The cells were washed with 1× PBS and the coverslips containing cells were mounted on slides and observed in confocal microscope (Olympus) under 60× oil immersion objective.

To validate the involvement of DNA damage-induced ncRNA in the formation of 53BP1 foci, 2 × 10^5^ HeLa cells were cotransfected with ShRNA targeting 3′-UTR of *SMARCAL1* along with vector alone or construct expressing wild-type *SMARCAL1* or construct expressing K464A mutant gene, or construct expressing R820H mutant gene. After 36 h, 2 μM doxorubicin was added in these cells for 10 min and ncRNA was isolated using mirVana microRNA Isolation Kit (Thermo Fisher Scientific, U.S.A.) according to the manufacturer’s instructions. The isolated ncRNA was stored at −20°C till further use.

For ncRNA put back experiment, HeLa cells were grown on coverslips and treated with 2 μM doxorubicin for 10 min. The cells were washed with 1× PBS four times followed by its permeabilization with 0.5% TritonX-100 in 1× PBS for 20 min at 4°C. The cells were again washed with 1× PBS and then treated with 1 mg/ml RNase for 20 min at room temperature. The cells were washed with 1× PBS 2–3 times and were incubated with 100 ng of ncRNA purified from the transfected cells as mentioned above for 1 h at room temperature. The cell were then washed with 1× PBS and processed for immunocytochemistry. The images were observed in confocal microscope (Olympus) under 60× oil immersion objective.

### RNA isolation and qPCR

Total RNA was extracted from both untreated and treated (2 μM doxorubicin for 10 min) HeLa cells after cotransfecting the cells with ShRNA against 3′ UTR region of *SMARCAL1* and vector alone or construct expressing wild-type *SMARCAL1* or construct expressing K464A mutant gene, or construct expressing R820H mutant gene. qPCR was performed as described previously [31]. The primers used for the qPCR experiments are provided in Supplementary Table S2.

## Results

The ATPase domain of the SWI2/SNF2 proteins consists of two RecA-like domains. Motifs I, Ia, II, and III are present in RecA-like domain 1 while motifs IV, V, and VI are present in RecA-like domain 2. Functional analysis in SWI2/SNF2 proteins has shown that motifs IV, V, and VI were important for ATP hydrolysis as well as for function [23]. However, it is not known whether these motifs mediate conformational integrity, or whether they are involved in ligand interaction or whether they are required for ATP hydrolysis. Therefore, the roles of motif IV, V, and VI present in the RecA-like domain 2 in interaction with ligands (DNA and ATP) in SWI2/SNF2 proteins using ADAAD as model system have been analyzed in the present study.

### The conserved phenylalanine of motif IV is required for both ATP and DNA binding in the presence of the other ligand

**Figure 1:**
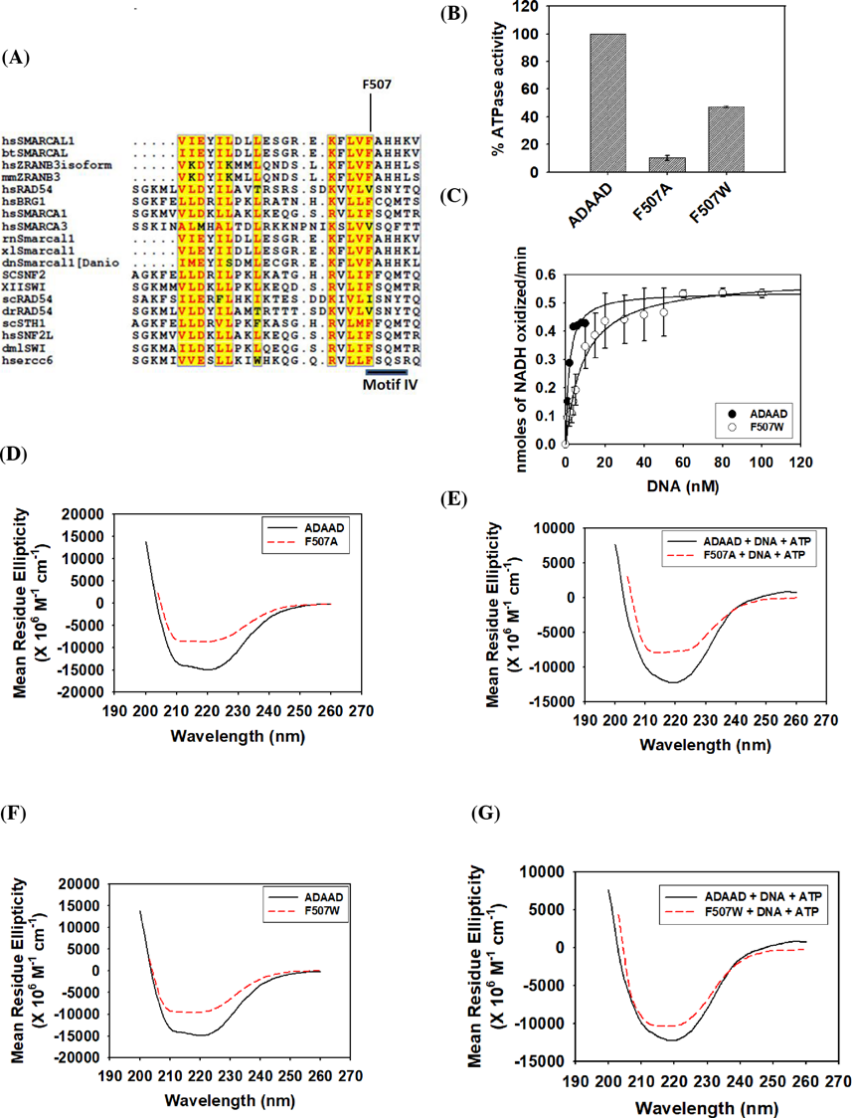
The conserved phenylalanine of motif IV is required for both ATP and DNA binding in the presence of the other ligand (**A**) Sequence alignment of the motif IV present in the SWI2/SNF2 proteins. Alignment was carried out using Clustal W program [41], and some minor editing was done manually. Highlighted box represents the conserved Motif IV, of which conserved phenylalanine residue was chosen for the mutation. (**B**) Comparison of ATPase activity of wild-type ADAAD, F507A, and F507W. (**C**) Comparison of kinetic data of F507W and wild-type ADAAD. (**D**) Comparison of CD spectra of F507A with the wild-type ADAAD in the absence of ligands. (**E**) Comparison of CD spectra of F507A with the wild-type ADAAD in the presence of ligands. (**F**) Comparison of CD spectra of F507W with wild-type ADAAD in the absence of ligands. (**G**) Comparison of CD spectra of F507W with wild-type ADAAD in the presence of ligands.

The motif IV of SWI2/SNF2 family contains a conserved phenylalanine residue (Figure 1A). Studies have shown that this motif is required for ATP-dependent binding of RNA substrates in SF2 helicases [32]. Therefore, the conserved phenylalanine (F507) in ADAAD was mutated to alanine as well as to tryptophan to understand the function of this aromatic residue in SWI2/SNF2 proteins. F507A was unable to hydrolyze ATP in the presence of stem–loop DNA, the preferred DNA effector for ADAAD; however, the ATPase activity was restored in case of F507W mutant underscoring the importance of the aromatic residue at position 507 (Figure 1B). However, tryptophan is not interchangeable with phenylalanine as calculation of the kinetic parameters showed that the *K*_M_ for F507W was 3.5-fold higher than for the wild-type protein (Figure 1C; Table 1). Further, though the *k*_cat_ did not change appreciably, the catalytic efficiency of F507W was 4-fold lower than that of the wild-type protein (Table 1).

**Table 1:** Calculation of kinetic parameters

Protein	*K*_M_ (nM)	*V*_max_ (µmol NADH oxidized/min)	*k*_cat_ (min^−1^)	*k*_cat_/*K*_M_ (nM^−1^ min^−1^)
**ADAAD**	1.8 ± 0.04	0.5 ± 0.0004	21.5 ± 0.02	11.7 ± 0.3
**F507W**	6.8 ± 1.8	0.6 ± 0.1	21.8 ± 4.0	3.0 ± 0.2
**S558A**	6.0 ± 0.4	0.7 ± 0.02	28.9 ± 0.7	4.8 ± 0.3
**T560A**	7.0 ± 0.6	0.8 ± 0.01	30.4 ± 0.3	4.3 ± 0.3
**H594A**	36.1 ± 4.5	0.5 ± 0.2	21.5 ± 8.5	0.59 ± 0.2

Data are presented as an average ± s.d. of two independent experiments with each experiment done in duplicates. In all these experiments, 0.1 μM of protein was used.

To understand whether structural variations between F507A, F507W, and the wild-type protein were responsible for the differences in the ability to hydrolyze ATP, the global structure was probed using CD spectroscopy. CD analysis of the purified F507A and F507W mutant showed that the structure of both the mutants was altered as compared with that of that of the wild-type protein both in the absence and presence of ligands, suggesting that the conserved phenylalanine was required for maintaining the secondary structure of the protein (Figure 1D–G) and cannot be replaced by another aromatic residue.

Next, the binding parameters were evaluated in order to understand the role of phenylalanine in ATP hydrolysis. Binding studies showed that F507A was able to bind to DNA in the absence of ATP with a *K*_d_ value similar to the wild-type protein but the interaction with this ligand in the presence of ATP was impaired by 10-fold as compared with ADAAD (Table 2; Supplementary Figure S3A). Similarly, the mutant protein was able to bind to ATP in the absence of DNA with a *K*_d_ value comparable to the wild-type protein but the interaction in the presence of DNA was 3-fold weaker as compared with ADAAD (Table 3; Supplementary Figure S3B). The binding of DNA in the presence of ATP in case of F507W was 3-fold weaker as compared with ADAAD suggesting that the interaction with DNA was partially restored in the presence of the aromatic residue (Table 2; Supplementary Figure S3C). The binding of ATP in the presence of DNA was largely restored in F507W (Table 3; Supplementary Figure S3D) leading us to hypothesize that the presence of an aromatic residue at this position in motif IV is critical for interaction with both ATP and DNA. Further, the presence of phenylalanine is needed for maintaining the conformation of the protein.

**Table 2:** *K*_d_ values for DNA binding with mutant proteins were calculated using one-site saturation model from the binding data

	*K*_d_ (M)	*R*^2^
	**Interaction with DNA in the absence of ATP**	
ADAAD*	(19.9 ± 4.9) × 10^−9^	0.96
**F507A**	(19.0 ± 4.5) × 10^−9^	0.98
**F507W**	(44.8 ± 9.0) × 10^−9^	0.96
**S558A**	(11.0 ± 2.7) × 10^−9^	0.99
**T560A**	(6.8 ± 0.6) × 10^−9^	0.96
**R592A**	(31.5 ± 1.3) × 10^−9^	0.98
**H594A**	(10.9 ± 2.6) × 10^−9^	0.99
**R595A**	(24.0 ± 0.6) × 10^−9^	0.96
**R595K**	(25.1 ± 7.4) × 10^−9^	0.91
**D591H**	(21.3 ± 7.0) X 10^−9^	0.96
**H329D/D591H**	(16.1 ± 5.7) × 10^−9^	0.96
**F507A/R592A**	(27.8 ± 3.6) × 10^−9^	0.95
**R595H**	(24.3 ± 6.1) × 10^−9^	0.94
	**Interaction with DNA in the presence of ATP**	
ADAAD*	(3.4 ± 0.2) × 10^−9^	0.97
**F507A**	(32.8 ± 6.0) X 10^−9^	0.98
**F507W**	(14.0 ± 2.2) × 10^−9^	0.98
**S558A**	(13.8 ± 2.2) × 10^−9^	0.92
**T560A**	(1.4 ± 0.3) × 10^−9^	0.97
**R592A**	(6.4 ± 1.1) × 10^−9^	0.92
**H594A**	(6.6 ± 0.9) × 10^−9^	0.95
**R595A**	(7.5 ± 1.5) × 10^−9^	0.96
**R595K**	(7.9 ± 2.1) × 10^−9^	0.87
**D591H**	(6.9 ± 1.4) × 10^−9^	0.90
**H329D/D591H**	(9.9 ± 2.2) X 10^−9^	0.90
**F507A/R592A**	(9.4 ± 0.9) × 10^−9^	0.92
**R595H**	(10.1 ± 0.3) × 10^−9^	0.93

Data are presented as an average ± s.d. of three binding curves.

* Reported in [24].

**Table 3:** *K*_d_ values for ATP binding with mutant proteins were calculated using one-site saturation model from the binding data

	*K*_d_ (M)	*R*^2^
	**Interaction with ATP in the absence of DNA**	
ADAAD*	(1.6 ± 0.5) × 10^−6^	0.98
**F507A**	(1.6 ± 0.4) × 10^−6^	0.98
**F507W**	(1.0 ± 0.3) × 10^−6^	0.98
**S558A**	(1.9 ± 0.4) × 10-6	0.95
**T560A**	(1.0 ± 0.3) × 10^−6^	0.99
**R592A**	(1.6 ± 0.6) × 10^−6^	0.98
**H594A**	(1.6 ± 0.2) × 10^−6^	0.98
**R595A**	(1.2 ± 0.2) × 10^−6^	0.96
**R595K**	(1.2 ± 0.2) × 10^−6^	0.97
**D591H**	(3.2 ± 0.3) × 10^−6^	0.97
**H329D/D591H**	(2.8 ± 0.1) × 10^−6^	0.98
**F507A/R592A**	(1.7 ± 0.1) × 10^−6^	0.99
**R595H**	(1.3 ± 0.2) × 10^−6^	0.98
	**Interaction with ATP in the presence of DNA**	
ADAAD*	(0.14 ± 0.03) × 10^−6^	0.98
**F507A**	(0.45 ± 0.02) × 10^−6^	0.99
**F507W**	(0.22 ± 0.03) × 10^−6^	0.99
**S558A**	(0.36 ± 0.005) × 10^−6^	0.98
**T560A**	(0.22 ± 0.03) × 10^−6^	0.99
**R592A**	(0.24 ± 0.02) × 10^−6^	0.97
**H594A**	(0.09 ± 0.01) × 10^−6^	0.99
**R595A**	(0.11 ± 0.02) × 10^−6^	0.97
**R595K**	(0.11 ± 0.04) × 10^−6^	0.98
**D591H**	(0.21 ± 0.06) × 10^−6^	0.99
**H329D/D591H**	(0.24 ± 0.02) × 10^−6^	0.96
**F507A/R592A**	(0.24 ± 0.04) × 10^−6^	0.98
**R595H**	(0.23 ± 0.07) × 10^−6^	0.95

Data are presented as an average ± s.d. of three binding curves.

* Reported in [24].

### Motif V determines the catalytic efficiency of the protein

**Figure 2:**
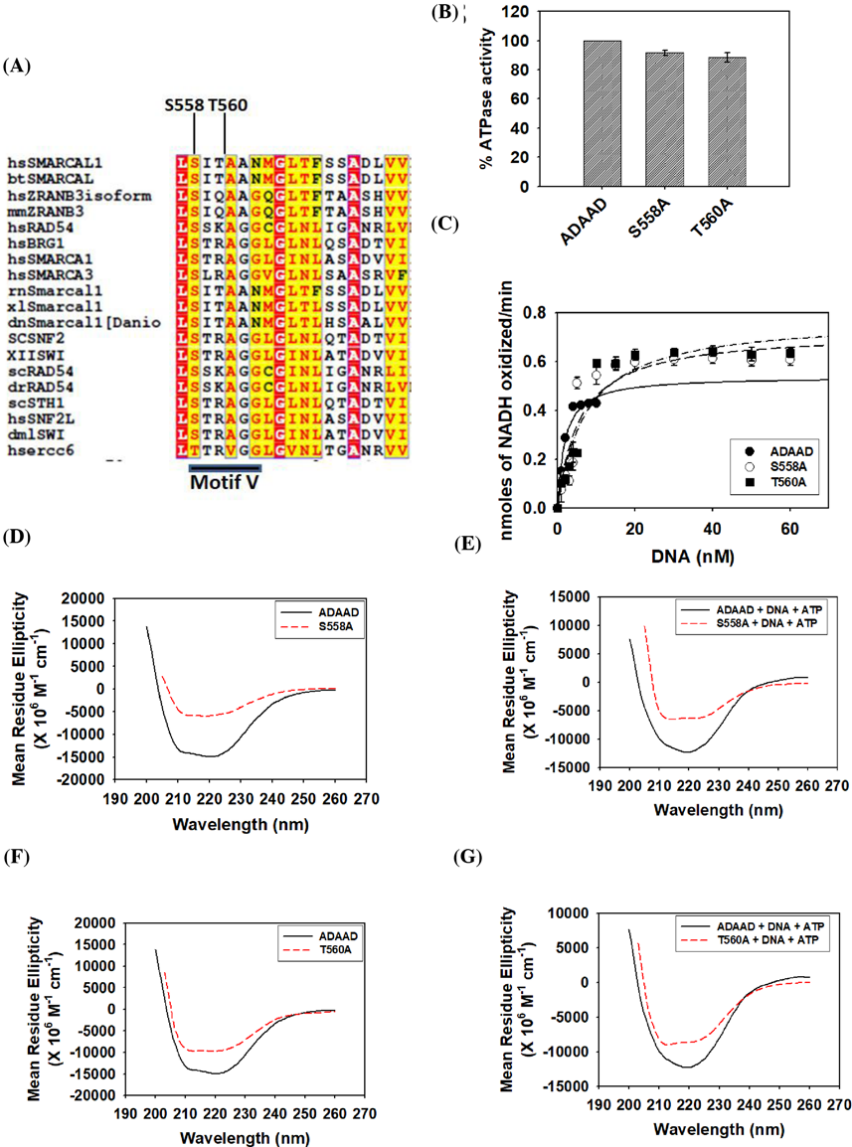
Motif V determines the catalytic efficiency of the protein (**A**) Sequence alignment of motif V in SWI2/SNF2 proteins using Clustal W software [41]. Serine (S558) and threonine (T560) of motif V were chosen for the study. (**B**) Comparison of the ATPase activity of the mutants with the wild-type protein. (**C**) Comparison of kinetic data of S558A and T560A with the wild-type ADAAD. (**D**) Comparison of CD spectra of S558A with wild-type ADAAD in the absence of ligands. (**E**) Comparison of CD spectra of S558A with the ADAAD in the presence of ligands. (**F**) Comparison of CD spectra of T560A with the wild-type protein in the absence of ligands. (**G**) Comparison of CD spectra of T560A with the wild-type protein in the presence of ligands.

Bioinformatic analysis enabled us to identify the conserved sequences of motif V. In SMARCAL1 family, this sequence consists of SITAAN as opposed to STRAAG sequence generally present in other SWI2/SNF2 members (Figure 2A). The serine (S558) and threonine (T560) of SITAAN sequence were mutated to alanine.

The purified mutant proteins, S558A and T560A, were able to hydrolyze ATP (Figure 2B); however, calculation of the kinetic parameters showed that the *K*_M_ for DNA was approximately 3-fold higher for the mutant proteins as compared with the wild-type protein (Figure 2C; Table 1). Though the *k*_cat_ was not significantly altered as compared with the wild-type protein, the catalytic efficiency of both the mutant proteins was approximately 3-fold lower than the wild-type protein, indicating the importance of the motif V residues in ATP hydrolysis (Table 1).

CD studies showed that the secondary structure of both S558A and T560A was altered as compared with the wild-type protein both in the absence and presence of ligands (Figure 2D–G). Binding parameters showed that S558A was able to bind to the DNA in the absence of ATP with an affinity comparable to the wild-type protein (Table 2; Supplementary Figure S4A). However, the interaction of this mutant protein with DNA in the presence of ATP was approximately 3-fold weaker as compared with the wild-type protein (Table 2; Supplementary Figure S4A). On the other hand, T560A was able to bind to DNA both in the absence and presence of ATP with a 2.5-fold higher affinity as compared with the wild-type protein (Table 2; Supplementary Figure S3B). Further, the interaction of both S558A and T560A with ATP in the absence of DNA was similar to that of the wild-type protein (Table 3). However, S558A interaction with ATP in the presence of DNA was 2.5-fold weaker while the interaction of T560A with ATP in the presence of DNA was similar to that of the wild-type protein (Table 3; Supplementary Figure S4C and D), suggesting that S558 but not T560 may be required for interaction with ATP in the presence of DNA.

Thus, based on these results, we conclude that S558 and T560 of motif V are required for interaction with DNA by maintaining the secondary structure conformation of the protein. S558A mutation results in a conformation that mediates weaker interaction with DNA in the presence of ATP while T560A mutation leads to a conformation that supports tighter interaction with DNA both in the absence and presence of ATP. In addition, S558 might also be dictating the interaction with ATP in the presence of DNA. Thus, mutations in S558 and T560 ultimately result in mutant proteins that hydrolyze ATP with altered kinetics.

### The conserved residues of motif VI are needed for conformational integrity and for interaction with DNA in the presence of ATP

**Figure 3:**
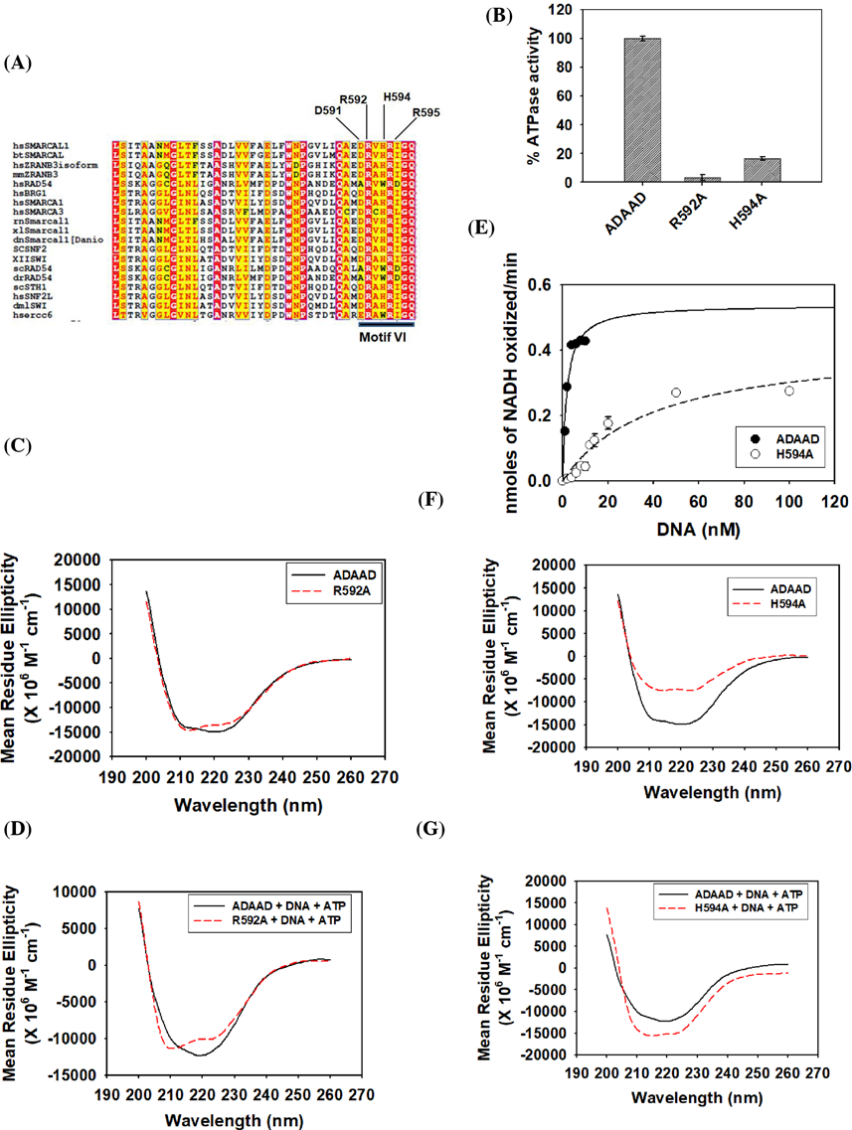
Biochemical characterization of motif VI mutants—R592A and H594A (**A**) Clustal W analysis of motif VI in SWI2/SNF2 proteins [41]. (**B**) Comparison of ATPase activity of R592A and H594A with wild-type ADAAD. (**C**) Comparison of the CD spectra of R592A with wild-type ADAAD in the absence of ligands. (**D**) Comparison of the CD spectra of R592A with wild-type ADAAD in the presence of ligands. (**E**) Comparison of kinetic data of H594A with the wild-type protein. (**F**) Comparison of the CD spectra of H594A with wild-type ADAAD in the absence of ligands. (**G**) Comparison of CD spectra of H594A with wild-type ADAAD in the presence of ligands.

Studies using eIF4A, a SF2 family RNA helicase, has shown that motif VI is required for both RNA binding and ATP hydrolysis [20]. This motif is also believed to mediate both interlobe and intralobe communication [21]. Bioinformatic analysis identified the conserved residues DRAHRIGQ of motif VI in SWI2/SNF2 proteins (Figure 3A). This analysis showed that two arginines (R592 and R595 in ADAAD) are absolutely conserved in the SWI2/SNF2 proteins (Figure 3A). It should be noted that these two arginines are also conserved in other SF1 and SF2 helicases [12].

The ATPase assay showed that the mutant R592A was unable to hydrolyze ATP (Figure 3B). CD spectroscopy showed that the structure of the protein was slightly altered in the absence of ligands and prominently altered in the presence of the ligands (Figure 3C,D). Binding studies showed that the interaction with DNA was unaltered in the absence of ATP but was 2-fold weaker in the presence of ATP (Table 2; Supplementary Figure S5A). Further, the interaction with ATP was unaltered in the absence and presence of DNA (Table 3; Supplementary Figure S5B). Thus, R592 is required only for maintaining the conformational integrity of the protein and the loss in ATPase activity can be solely attributed to the weaker interaction with DNA in the presence of ATP due to loss in secondary structure conformation.

Next, the role of H594 was analyzed. The mutant showed 20% residual ATPase activity (Figure 3B); however, the kinetic parameters indicate that both the *K*_M_ and the *V*_max_ were altered resulting in a catalytically inefficient protein (Figure 3E; Table 1). The protein conformation as compared with the wild-type protein was altered both in the absence and presence of ligands (Figure 3F,G). H594–DNA interaction was unaltered as compared with wild-type ADAAD–DNA interaction in the absence of ATP (Table 2; Supplementary Figure S5C). However, the interaction between the mutant protein and DNA was 2-fold weaker as compared with ADAAD–DNA interaction in the presence of ATP (Table 2; Supplementary Figure S5C). The H594A–ATP interaction was similar to the wild-type ADAAD–ATP interaction both in the absence and presence of DNA (Table 3; Supplementary Figure S5D). Thus, the altered conformation after H594A mutation results in weaker protein–DNA interaction in the presence of ATP that possibly leads to catalytically inefficient protein.

**Figure 4:**
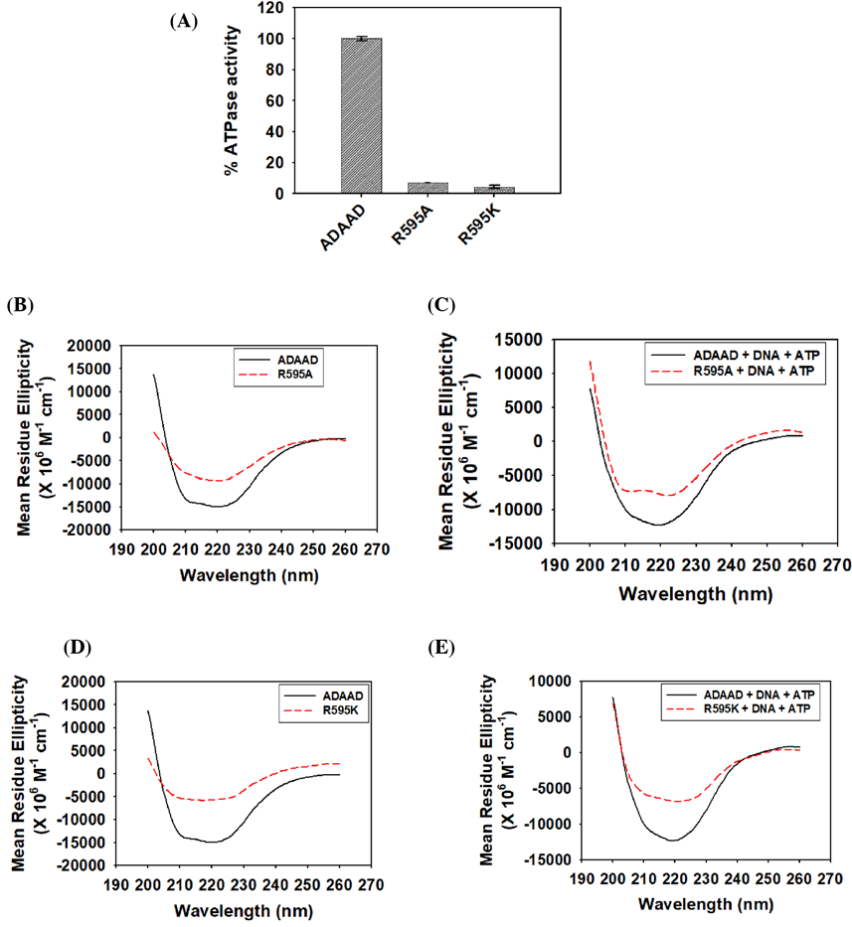
Arginine 595 of motif VI is required for maintaining the secondary structure of the protein as well as for interaction with DNA in the presence of ATP (**A**) Comparison of the ATPase activity of R595A and R595K with wild-type ADAAD. (**B**) Comparison of the CD spectra of R595A with wild-type ADAAD in the absence of ligands. (**C**) Comparison of the CD spectra of R595A with wild-type ADAAD in the presence of ligands. (**D**) Comparison of the CD spectra of R595K with wild-type ADAAD in the absence of ligands. (**E**) Comparison of CD spectra of R595K with wild-type ADAAD in the presence of ligands.

Next, R595 was mutated to a neutral amino acid alanine (R595A) as well as to positively charged amino acid lysine (R595K). Both R595A and R595K were unable to hydrolyze ATP (Figure 4A). CD analysis showed that the conformation of the two mutants was altered with respect to the wild-type protein both in the absence and presence of ligands, suggesting that the loss in ATPase activity could be due to altered protein conformation (Figure 4B–E).

To understand whether the loss in the secondary structure conformation resulted in altered interaction with ligands, binding studies were performed. The interaction of the arginine mutant proteins with DNA was unchanged as compared with the wild-type protein in the absence of ATP (Table 2; Supplementary Figure S6A and C). Further, in the presence of ATP, DNA binding was 2-fold less than the wild-type protein in case of both R595A and R595K (Table 2; Supplementary Figure S6A and C). However, the mutant proteins were able to bind to ATP in the absence and presence of DNA with the same *K*_d_ as the wild-type protein (Table 3; Supplementary Figure S6B and D), once again indicating that the conformational change in the mutant proteins results in impaired interaction with DNA in the presence of ATP possibly leading to loss in ATPase activity.

### Motif VI and motif II possibly do not form a salt bridge

The formation of a salt bridge between the conserved histidine of motif VI and aspartate of motif II has been shown in SF2 helicases [21]. This salt bridge is important for interlobe communication and consequently, for the function. In case of SWI2/SNF2 proteins, the histidine present in motif VI present in eIF4A is replaced by aspartate. Further, the last aspartate of motif II (DEAD) is replaced by a histidine (DEXH) in SWI2/SNF2 proteins (Supplementary Figure S7). In the crystal structure of SsoRad54, the RecA-domain 2 is flipped 180° in comparison with other helicases [9]. We hypothesized that in the absence of ligands, due to the flipping of the domains, salt bridge formation between motif VI and II is not possible. However, if salt bridge formation is important for ATP hydrolysis as in the case of other helicases, then the aspartate of motif VI and histidine of motif II might interact in the presence of the ligands (Supplementary Figure S7).

**Figure 5:**
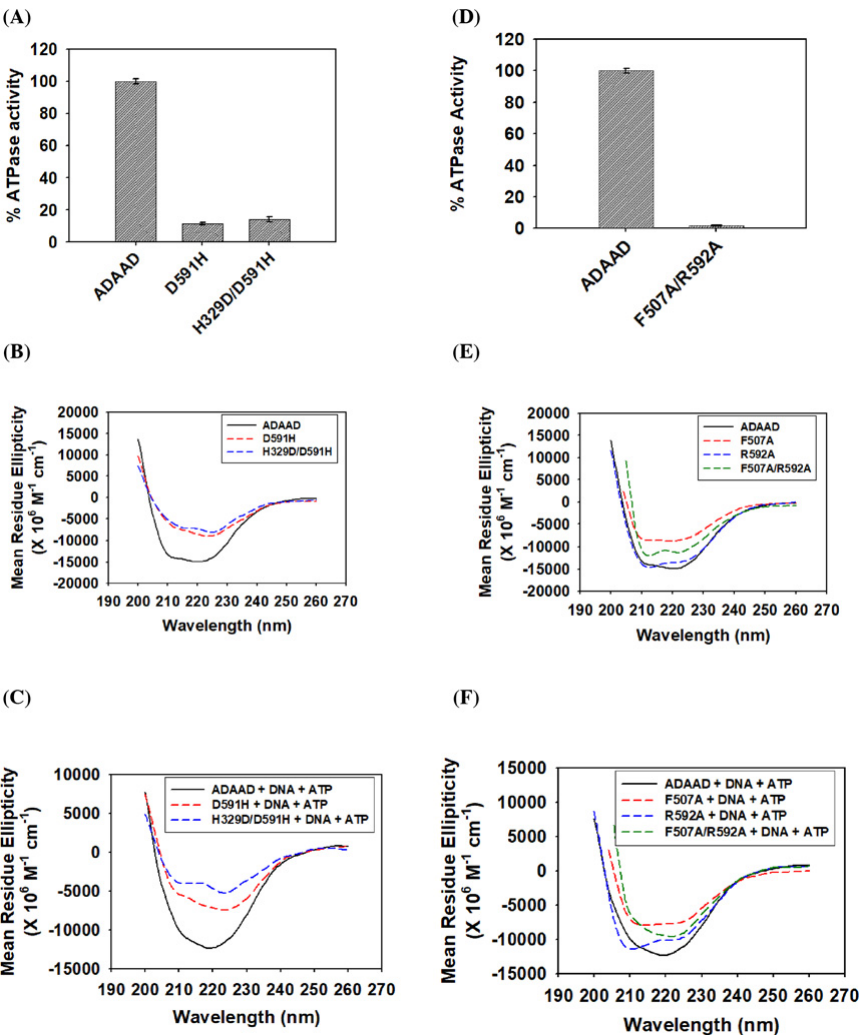
Studying the interlobe and intralobe communication (**A**) Comparison of the ATPase activity of D591H and H329D/D591H with wild-type ADAAD. (**B**) Comparison of the CD spectra of D591H and H329D/D591H with the wild-type ADAAD in the absence of ligands. (**C**) Comparison of the CD spectra of D591H and H329D/D591H with the wild-type ADAAD in the presence of ligands. (**D**) Comparison of the ATPase activity of F507A/R592A with wild-type ADAAD. (**E**) Comparison of the CD spectra of F507A, R592A, and F507A/R592A with wild-type ADAAD in the absence of ligands. (**F**) Comparison of the CD spectra of F507A, R592A, and F507A/R592A with wild-type ADAAD in the presence of ligands.

A single mutant D591H as well as the double mutant H329D/D591H in ADAAD was created where D591 is present in motif VI while H329 is present in motif II and as per the hypothesis, these residues should be forming a salt bridge. Further, D591H should not be able to hydrolyze ATP but the ATPase activity should be restored in the case of the double mutant H329D/D591H. As expected, D591H was unable to hydrolyze ATP (Figure 5A). Contrary to our expectation, H329D/D591H also did not possess ATPase activity (Figure 5A). CD analysis showed that the structure of the mutant proteins was altered as compared with the wild-type protein both in the absence and presence of the ligands (Figure 5B,C). Binding analysis showed that the interaction with DNA was unaltered in the absence of ATP; however, DNA binding was impaired in both D591H as well as with the double mutant H329D/D591H in the presence of ATP (Table 2 and Supplementary Figure S8A and C). Moreover, ATP binding was unchanged for both D591H and H329D/D591H mutant in the absence and presence of DNA (Table 3 and Supplementary Figure S8B and D). From these results we hypothesized that D591 was required for maintaining structural conformation and possibly for interaction with DNA in the presence of ATP. As the conformation as well as ATPase activity was not restored in the double mutant, we could not find any evidence that D591 makes a salt bridge with H329 in ADAAD either in the absence or presence of ligands.

### F507A/R592A double mutant shows that the residues of motif VI play a more important role in ADAAD

In the crystal structures of SF2 helicases, the conserved phenylalanine of motif IV has been shown to stack on the conserved arginine of motif IV. In Ded1, the phenylalanine of motif IV has been shown to play a more important role than arginine [32].

The stacking interaction in case of SWI2/SNF2 proteins has not been reported in the available crystal structure [7,9]. Therefore, to probe whether this interaction exists in ADAAD, F507 (motif IV) and R592 (motif VI) were mutated to create a double mutant as these residues correspond to the residues reported to form cation–π interaction [32].

The double mutant F507A/R592A was also unable to hydrolyze ATP (Figure 5D). The secondary structure analysis showed that the structure of the mutant protein F507A/R592A was altered as compared with the wild-type protein both in the absence and presence of the ligands (Figure 5E,F). Binding studies showed that the interaction with DNA in the absence of ATP in the double mutant F507A/R592A was similar to the wild-type protein but the interaction with the DNA in the presence of ATP was approximately 3-fold weaker as compared with the wild-type protein (Table 2). This interaction was approximately similar to the interaction of R592A with DNA in the presence of ATP, suggesting that R592 might be the critical determinant in the interaction with DNA in the presence of ATP (Table 2 and Supplementary Figure S8E). A similar result was obtained when the interaction with ATP in the presence of DNA was studied wherein the interaction parameters of F507A/R592 was comparable to R592A–ATP interaction (Table 3 and Supplementary Figure S8F). Thus, in ADAAD, unlike Ded1, the arginine of motif VI appears to dictate the conformation of the protein as well as its interaction with the ligands.

### Mutations in RecA-like domain 2 do not alter the conformational stability of the protein

**Figure 6:**
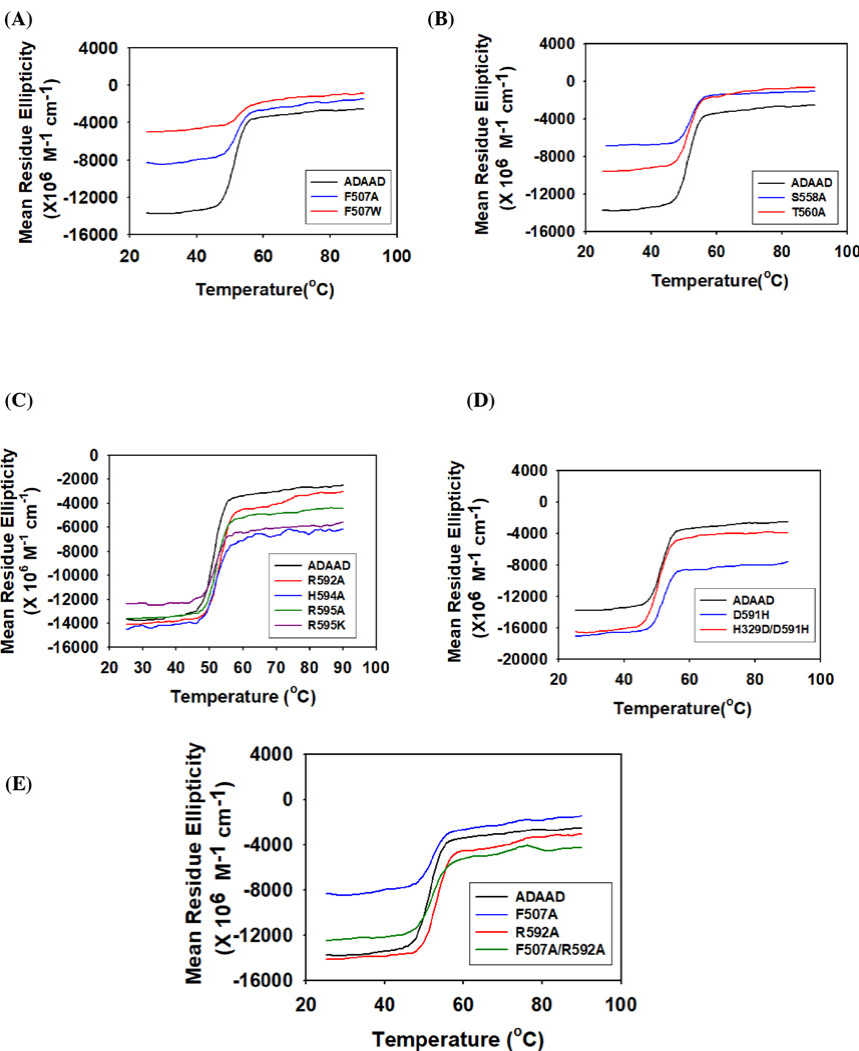
Mutations in RecA-like domain 2 do not alter the conformational stability of the protein The thermal stability of the mutants was compared with wild-type ADAAD. (**A**) Motif IV mutants F507A and F507W, (**B**) motif V mutants S558A and T560A, (**C**) motif VI mutants R592A, H594A, R595A and R595K, (**D**) D591H and H329D/D591H, (**E**) F507A, R592A and F507A/R592A.

One of the overwhelming features of all the mutations presented till now is the alteration in the conformation of the protein in the absence of the ligand. The question that we addressed next was whether these mutations alter the stability of the protein or whether they impact only the conformational integrity. To answer this question, thermal denaturation studies were performed with all the mutants. The present study showed that the *T*_m_ of the mutant protein was similar to the wild-type protein indicating that the conformational stability of the proteins has not been impacted by the mutations (Figure 6). Thus, the conserved residues present in motifs IV, V, and VI are involved only in maintaining the conformational integrity and not the conformational stability.

### The DNA damage response pathway is impaired in the SIOD-associated mutant R820H

Mutations in SMARCAL1 is associated with SIOD. The mutation R820H has been found in patients with SIOD [29]. This mutation maps to motif VI and the residue R820 corresponds to R595 of ADAAD. To understand how mutation of arginine to histidine affects ATPase activity of the protein, R595H mutation was made in ADAAD.

**Figure 7:**
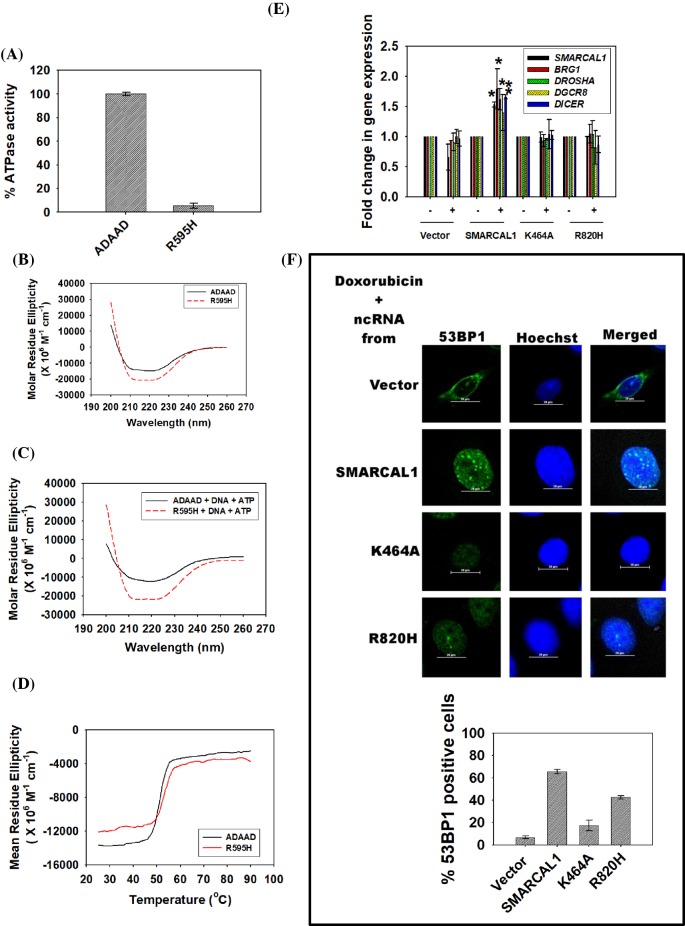
DNA damage response is impaired in SIOD-associated mutant, R820H (**A**) Comparison of ATPase activity of R595H with wild-type protein. (**B**) Comparison of CD spectra of R595H with wild-type ADAAD in the absence of ligands. (**C**) Comparison of CD spectra of R595H with wild-type ADAAD in the presence of ligands. (**D**) Comparison of the conformational stability of the mutant, R595H, with wild-type ADAAD. (**E**) The transcript levels of *SMARCAL1, BRG1, DROSHA, DGCR8*, and *DICER* were estimated using qPCR. *GAPDH* was used as internal control. Star indicates significance at *P*<0.05. (**F**) The formation of 53BP1 foci was monitored in HeLa cells after treatment with 2 μM doxorubicin for 10 min. After inducing DNA damage, cells were treated with RNase as explained in Methods section. The RNase was removed and ncRNA purified from HeLa cells cotransfected with shRNA against 3′-UTR of *SMARCAL1* along with either vector alone, or construct expressing wild-type *SMARCAL1*, or construct expressing K464A mutant or construct expressing R820H mutant, was added. Prior to isolation of ncRNA, the transfected cells were treated with 2 μM doxorubicin for 10 min. The 53BP1 foci formation was observed using confocal microscope and the number of cells containing ≥ 5 foci/cell was quantitated. The data are presented as an average ± s.d for two independent experiments where > 100 cells were counted in each experiment.

The mutated protein was unable to hydrolyze ATP (Figure 7A) and CD spectroscopy showed that the secondary structure of the mutant protein was altered as compared with the wild-type protein both in the absence and presence of ligands (Figure 7B,C). Interestingly, while the secondary structure of wild-type ADAAD was changed in the presence of ligands (Supplementary Figure S9A), the secondary structure of the mutant protein was similar both in the absence and presence of ligands indicating that the conformational change required for ATP hydrolysis did not happen when R595 was mutated to histidine (Supplementary Figure S9B). Thermal denaturation experiment showed that the conformational stability of the mutant protein was similar to that of the wild-type protein (Figure 7D).

Binding studies showed that the interaction of DNA in the presence of ATP was altered as compared with the wild-type protein indicating that the altered secondary structure possibly results in weaker interaction with DNA in the presence of ATP (Tables 2 and 3; Supplementary Figure S9C and D). Further, the conformational change associated with ligand binding does not occur in R595H mutant, thus resulting in an inactive protein.

SMARCAL1 mediates DNA damage response by modulating the expression of *BRG1* as well as of the RNAi genes (*DROSHA, DGCR8*, and *DICER*) on doxorubicin-induced double-strand breaks [33]. The up-regulation of RNAi genes results in synthesis of noncoding RNA (ncRNA) that is required for the formation of 53BP1 foci [33]. We, therefore, asked how R820H mutation in SMARCAL1 affects the 53BP1 foci formation on doxorubicin-induced double-strand breaks. The mutation R820H was introduced into the human *SMARCAL1* gene using site-directed mutagenesis. HeLa cells were transfected with ShRNA directed against the 3′-UTR of *SMARCAL1* so that the endogenous production of SMARCAL1 was down-regulated. These cells were cotransfected either with vector alone or with construct expressing wild-type *SMARCAL1* or construct expressing K464A mutant gene [30], or construct expressing R820H mutant gene. After 36 h, cells were treated with doxorubicin for 10 min and the expression of *SMARCAL1, BRG1*, and the RNAi genes were evaluated. The expression of the genes was up-regulated only when HeLa cells were cotransfected with ShRNA and wild-type construct implying that the mutated gene was not able to mediate transcriptional regulation of these genes (Figure 7E). The K464A mutant was used as control as it has been previously shown to be defective in ATPase activity [24,30].

Next, the ability to form ncRNA for the formation of 53BP1 foci was studied. HeLa cells were treated with doxorubicin for 10 min and 53BP1 foci were observed by immunochemistry (Supplementary Figure S9E) [34]. The foci were abolished if treated with RNase indicating RNA was critical for the formation of the foci (Supplementary Figure S9E) [34]. The foci formation was restored if ncRNA purified from cells cotransfected with ShRNA along with wild-type *SMARCAL1* construct, indicating SMARCAL1 was able to support the formation of 53BP1 foci (Figure 7F). However, ncRNA purified from cells transfected with ShRNA along with vector alone or from cells transfected with ShRNA along with either the gene carrying the K464A mutant or R820H mutant was unable to restore the formation of 53BP1 foci, indicating DNA damage response was impaired when cells carried this mutation (Figure 7F).

## Discussion

The SWI2/SNF2 proteins contain an ATPase domain that mediates hydrolysis of ATP. Structurally, this domain is further subdivided into RecA-like domain 1 and RecA-like domain 2 [7,9]. Previously we had shown that the amino acid residues present in motifs of RecA-like domain 1 of bovine homolog of SMARCAL1, ADAAD, are required for interaction of the protein with DNA, and thus, for ATP hydrolysis [25]. The functional importance of the motifs present in the RecA-like domain 2 has not yet been delineated and therefore, we have focused on the characterization of motifs IV, V, and VI present in this domain.

The crystal structure of *Sulfolobus solfatricus* Rad54 has shown that the RecA-like domain 2 in the SWI2/SNF2 proteins is needed either for ATPase activity or for maintaining the proper orientation of the protein in the presence of ATP. Thus, mutations in this domain are hypothesized to either impair ATPase activity or prevent the correct orientation of the protein in the presence of ATP to allow for ATP hydrolysis [9]. Further, in case of DEAD protein, it has been proposed that the residues of the domain 2 possibly couple the conformational changes on ATP binding to RNA binding [35].

The biochemical results presented in the present study show that the three motifs present in the RecA-like domain 2 are indeed essential for the interaction with DNA in the presence of ATP as well as for structural integrity. Interestingly, the mutations in these motifs do not impact the stability of the protein. Thus, the conserved residues present in RecA-like domain 2 appear to function as a molecular anchor to maintain the conformation of the protein without affecting the stability/folding of the protein. Further, as in the case of DEAD-box containing helicases, the motifs present in this domain seem to couple the conformational changes that occur on ATP binding to DNA binding.

The motif IV contains a conserved phenylalanine residue preceded by two hydrophobic residues in SF2 helicases. Though, the motif IV of SF2 helicases been shown to be important for DNA-stimulated ATPase activity in Rad54 from *Sulfolobus solfatricus* [9], the role of the phenylalanine or the other conserved hydrophobic residues have not be characterized in these proteins. This conserved phenylalanine in Ded1 protein, which belongs to the SF2 subfamily RNA helicases, has been shown to be important for RNA binding in the presence of ATP, thus, linking RNA binding to ATP hydrolysis [32]. However, in Ded1, the phenylalanine cannot be replaced by tryptophan, indicating preference not for aromaticity but for a particular amino acid at this position [32]. In contrast, in ADAAD, the presence of an aromatic residue in motif IV at position 507 is essential for ATP hydrolysis. Thus, the phenylalanine could be replaced with tryptophan yielding a catalytic active protein albeit with altered kinetics. Previously, we had shown that the interaction of ADAAD with ATP was through the adenine moiety [25]. Based on the biochemical data presented in the present study, we propose that the interaction of ATP in the presence of DNA with the protein might involve π–π interaction between the adenine ring of ATP and phenylalanine. However, we cannot rule out an indirect interaction as the motifs I, Ia, II, and III have been also shown to be sufficient for interaction of ATP with the protein both in the absence and presence of DNA [25].

The motif V has been shown to interact with the sugar-phosphate backbone of nucleic acids in UvrB, UL5, and NS3 proteins [18,36,37]. Further, in UL5, the motif V has been shown to be important for ATPase activity; mutations reduced but did not abolish the activity of the protein [36]. The importance of this motif has also been studied in Snf2 protein where deletion of motif V was shown to impact viability of yeast cells [23]. In contrast with these studies, in ADAAD, mutations in motif V result in approximately 3-fold higher *K*_M_ (weaker interaction with DNA) and 3-fold lower catalytic efficiency. The weaker interaction as observed by the *K*_M_ values was corroborated by the *K*_d_ values in case of S558A, suggesting that this residue is important for interaction with DNA in the presence of ATP. However, in case of T560A, the *K*_d_ data show that the interaction of the mutant protein with DNA is approximately 3-fold tighter as compared with the wild-type protein–DNA interaction both in the absence and presence of ATP, suggesting that this residue is critical for determining the rate of catalysis. Thus, in ADAAD, motif V is important both for interaction with DNA as well as for catalysis, possibly by maintaining the secondary structure of the protein. It needs to be noted that in the previous studies the motif V was deleted [23]. In addition, there is considerable difference between the amino acids present in motif V of SMARCAL1/ADAAD as compared with other family members (see Figure 2A). Thus, the variations observed between Snf2p and ADAAD could be a combination of these two factors.

The positively charged residues of motif VI are important for the structural integrity of the protein. The loss of conformation results in weaker interaction with DNA in the presence of ATP and therefore, loss of ATPase activity.

The biochemical studies performed in SF2 helicase members like UvrB, HCV, and eIF4A have shown a salt bridge formation between motif VI present in RecA-like domain 2A and motif II present in RecA-like domain 1A [17,21,38]. In these RNA helicases the first amino acid of motif VI, glutamine in case of UvrB and HCV and histidine in case of eIF4A, forms a salt bridge with the second aspartate of motif II. These residues are termed as “gatekeepers” [22]. However, such a salt bridge could not be detected in ADAAD as the double mutant H329D/D591H did not show any ATPase activity. This could be due to the fact that the RecA-like domain 2 is flipped out 180° in comparison with other helicases and the orientation in the presence of the ligands does not permit any interaction between the two domains [9]. It is also possible that the alterations caused by mutation in RecA-like domain 2 is not compensated by a mutation in RecA-like domain 1 and therefore, the double mutant protein possesses an altered secondary structure that precludes the formation of the salt bridge. This type of behavior has been reported for NS3 protein from Hepatitis C virus where similar double mutant was unable to hydrolyze ATP [22]. However, it should be noted that our studies just indicate that D591 and H329 are not forming a salt bridge; it is possible that other residues of the motif or other motifs are involved in this type of interaction.

The intralobe communication has been predicted to involve cation–π interaction particularly between the phenylalanine residue of motif IV and arginine residue of motif VI [32]. With Ded1, it was observed that the phenylalanine was playing a more important role than the arginine [32]. In contrast, our studies suggest that the arginine of motif VI plays a more important role as the double mutant shows the characteristics of the arginine mutant rather than the phenylalanine mutant.

Our biochemical studies indicate that the interaction of DNA with ADAAD is mediated by all the motifs present in the two RecA-like domains, thus, confirming the structural studies done with SF2 helicases. However, none of the conserved helicase residues, except F507 and S558, appear to be essential for interaction with ATP. It is possible that multiple motifs are involved in the interaction and thus, simultaneous disruption might yield information about the residues making contacts with ATP. In case of F507 present in motif IV, a 3-fold decrease in ATP binding could be observed in the presence of DNA indicating that this residue is directly involved via π–π interaction or indirectly involved in binding to ATP corroborating the studies done with DNA helicases [39,40].

Thus, the biochemical studies presented in the present study highlight the similarities and differences observed between ADAAD and other DNA/RNA helicases. Mutations in the human homolog of ADAAD, SMARCAL1, is associated with Schimke Immuno-Osseous Dysplasia wherein many of the mutations have been mapped to the Rec A-like domain 2 [29]. In particular, one of the mutations, R820H, corresponds to R595 present in motif VI of ADAAD. Our results show that the R820H mutant was unable to support DNA damage response as the ATPase activity of the protein was impaired and therefore, the ability to synthesize ncRNA required for 53BP1 foci formation was affected. Thus, our studies provide a biochemical basis to the pathophysiology associated with mutations in SMARCAL1.

## Supporting information

**supplementary Figure 1:** Purification of the wild type and mutant proteins. (A). Motif IV mutant proteins- F507A and F507- and motif V mutant proteins-S558A and T560A. (B) Motif VI mutant proteins: D591H, H594A, R592A, R595A, R595K, R595H and H329D/D591H. (C) Wild type ADAAD, F507A/R592A and R592A.

**supplementary Figure 2:** Representative fluorescence spectra of ADAAD titrated with (A). ATP; (B) DNA.

**supplementary Figure 3:** Interaction of F507A and F507W with ligands was studied using fluorescence spectroscopy. (A) Interaction of F507A with DNA in the absence and presence of saturating concentration of ATP. (B) Interaction of F507A with ATP in the absence and presence of saturating concentration of DNA. (C) Interaction of F507W with DNA in the absence and presence of saturating concentration of ATP. (D) F507W with ATP in the absence and presence of saturating concentration of DNA. All the titrations were done using fluorescence spectroscopy and represent the average ± standard deviation of two independent experiments. The protein concentration used in these experiments was 0.5 μM. was All the data were fitted using one-site saturation model.

**Supplementary Figure 4:** Interaction of S558A and T560A with ligands was studied using fluorescence spectroscopy. (A) Interaction of S558A with DNA in the absence and presence of saturating concentration of ATP. (B) Interaction of T560A with DNA in the absence and presence of saturating concentration of ATP. (C) Interaction of S558A with ATP in the absence and presence of saturating concentration of DNA. (D) Interaction of T560A with ATP in the absence and presence of saturating concentration of DNA. All the titrations were done using fluorescence spectroscopy and represent the average ± standard deviation of two independent experiments. The protein concentration used in these experiments was 0.5 μM. All the data were fitted using one-site saturation model.

**Supplementary Figure 5:** Interaction of R592A and H594A with the ligands was studied using fluorescence spectroscopy. (A) Interaction of R592A with DNA in the absence and presence of saturating concentration of ATP. (B) Interaction of R592A with ATP in the absence and presence of saturating concentration of DNA. (C) Interaction of H594A with DNA in the absence and presence of saturating concentration of ATP. (D) Interaction of H594A with ATP in the absence and presence of saturating concentration of DNA. All the titrations were done using fluorescence spectroscopy and represent the average ± standard deviation of two independent experiments. The protein concentration used in these experiments was 0.5 μM. All the data were fitted using one-site saturation model.

**Supplementary Figure 6:** Binding affinities were calculated of R595A and R595K for interaction with ATP and DNA using fluorescence spectroscopy. (A) Interaction of R595A with DNA in the absence and presence of saturating concentration of ATP. (B) Interaction of R595A with ATP in the absence and presence of saturating concentration of DNA. (C) Interaction of R595K with DNA in the absence and presence of saturating concentration of ATP. (D) Interaction of R595K with ATP in the absence and presence of saturating concentration of DNA. All the titrations were done using fluorescence spectroscopy and represent the average ± standard deviation of two independent experiments. The protein concentration used in these experiments was 0.5 μM. All the data were fitted using one-site saturation model.

**Supplementary Figure 7:** (A) Diagrammatic representation depicting inter-lobe communication in eIF4 [1] (B) In ADAAD the covariance in the positions of aspartate and histidine is observed leading us to ask whether a possible salt bridge exists between motif II and motif VI. (C) To test the hypothesis, the aspartate (D591) present in motif VI was mutated to histidine which resulted in abrogation of ATPase activity. (D) The ATPase activity was not restored when the histidine (H329) present in motif II was mutated to aspartate.

**Supplementary Figure 8:** The inter-lobe and intra-lobe interactions were studied using fluorescence spectroscopy. (A) Interaction of D591H with DNA in the absence and presence of saturating concentration of ATP. (B) Interaction of D591H with ATP in the absence and presence of saturating concentration of DNA. (C) Interaction of H329D/D591H with DNA in the absence and presence of saturating concentration of ATP. (D) Interaction of H329D/D591H with ATP in the absence and presence of saturating concentration of DNA. (E) Interaction of F507A/R592A with DNA in the absence and presence of saturating concentration of ATP. (F) Interaction of F507A/R592A with ATP in the absence and presence of saturating concentration of DNA. All the titrations were done using fluorescence spectroscopy and represent the average ± standard deviation of two independent experiments. The protein concentration used in these experiments was 0.5 μM. All the data were fitted using one-site saturation model.

**Supplementary Figure 9:** The DNA damage response pathway is impaired in the SIOD-associated mutant R820H. (A). Comparison of the CD spectra of wild type ADAAD in the absence and presence of ligands. (B). Comparison of the CD spectra of R595H in the absence and presence of ligands. (C). Interaction of R595H with DNA in the absence and presence of saturating concentration of ATP. (D). Interaction of R595H with ATP in the absence and presence of saturating concentration of DNA. All the titrations were done using fluorescence spectroscopy and represent the average ± standard deviation of two independent experiments. The protein concentration used in these experiments was 0.5 μM. All the data were fitted using one-site saturation model. (E) Formation of 53BP1 was monitored in HeLa cells after treatment with 2 μM doxorubicin for 10 min in the absence and presence of RNase. This data has been reported in [2] and is presented here to show the alteration in number of 53BP1 positive cells when HeLa cells are treated with doxorubicin and RNase.

**Supplemental Table 1:** List of primers used for making site-directed mutants

**Supplemental Table 1:** Primers used for qPCR.

## Abbreviations

ADAAD: Active DNA-dependent ATPase A Domain
SIOD: Schimke Immuno-Osseous Dysplasia

## References

[1] Hargreaves D.C. and Crabtree G.R. (2011) ATP-dependent chromatin remodeling: genetics, genomics and mechanisms. Cell Res. 21, 396–420 10.1038/cr.2011.32 21358755 PMC3110148

[2] Vincent J.A., Kwong T.J. and Tsukiyama T. (2008) ATP-dependent chromatin remodeling shapes the DNA replication landscape. Nat. Struct. Mol. Biol. 15, 477–484 10.1038/nsmb.1419 18408730 PMC2678716

[3] Kingston R.E. and Narlikar G.J. (1999) ATP-dependent remodeling and acetylation as regulators of chromatin fluidity. Genes Dev. 13, 2339–2352 10.1101/gad.13.18.2339 10500090

[4] Ho L. and Crabtree G.R. (2010) Chromatin remodelling during development. Nature 463, 474–484 10.1038/nature08911 20110991 PMC3060774

[5] Flaus A. and Owen-Hughes T. (2011) Mechanisms for ATP-dependent chromatin remodelling: the means to the end. FEBS J. 278, 3579–3595 10.1111/j.1742-4658.2011.08281.x 21810178 PMC4162296

[6] Gorbalenya A.E. and Koonin E.V. (1993) Helicases: amino acid sequence comparisons and structure-function relationships. Curr. Opin. Struct. Biol. 3, 419–429 10.1016/S0959-440X(05)80116-2

[7] Thomä N.H., Czyzewski B.K., Alexeev A.A., Mazin A.V., Kowalczykowski S.C. and Pavletich N.P. (2005) Structure of the SWI2/SNF2 chromatin-remodeling domain of eukaryotic Rad54. Nat. Struct. Mol. Biol. 12, 350–356 10.1038/nsmb919 15806108

[8] Hauk G., McKnight J.N., Nodelman I.M. and Bowman G.D. (2010) The chromodomains of the Chd1 chromatin remodeler regulate DNA access to the ATPase motor. Mol. Cell 39, 711–723 10.1016/j.molcel.2010.08.012 20832723 PMC2950701

[9] Dürr H., Körner C., Müller M., Hickmann V. and Hopfner K.-P. (2005) X-ray structures of the Sulfolobus solfataricus SWI2/SNF2 ATPase core and its complex with DNA. Cell 121, 363–373 10.1016/j.cell.2005.03.026 15882619

[10] Flaus A., Martin D. M.A., Barton G.J. and Owen-Hughes T. (2006) Identification of multiple distinct Snf2 subfamilies with conserved structural motifs. Nucleic Acids Res. 34, 2887–2905 10.1093/nar/gkl295 16738128 PMC1474054

[11] Dürr H., Flaus A., Owen-Hughes T. and Hopfner K.-P. (2006) Snf2 family ATPases and DExx box helicases: differences and unifying concepts from high-resolution crystal structures. Nucleic Acids Res. 34, 4160–4167 10.1093/nar/gkl540 16935875 PMC1616948

[12] Fairman-Williams M.E., Guenther U.-P. and Jankowsky E. (2010) SF1 and SF2 helicases: family matters. Curr. Opin. Struct. Biol. 20, 313–324 10.1016/j.sbi.2010.03.011 20456941 PMC2916977

[13] Velankar S.S., Soultanas P., Dillingham M.S., Subramanya H.S. and Wigley D.B. (1999) Crystal structures of complexes of PcrA DNA helicase with a DNA substrate indicate an inchworm mechanism. Cell 97, 75–84 10.1016/S0092-8674(00)80716-3 10199404

[14] Sinha K.M., Glickman M.S. and Shuman S. (2009) Mutational analysis of Mycobacterium UvrD1 identifies functional groups required for ATP hydrolysis, DNA unwinding, and chemomechanical coupling. Biochemistry 48, 4019–4030 10.1021/bi900103d 19317511 PMC2761027

[15] Papanikou E., Karamanou S., Baud C., Sianidis G., Frank M. and Economou A. (2004) Helicase Motif III in SecA is essential for coupling preprotein binding to translocation ATPase. EMBO Rep. 5, 807–811 10.1038/sj.embor.7400206 15272299 PMC1299117

[16] Dillingham M.S., Soultanas P. and Wigley D.B. (1999) Site-directed mutagenesis of motif III in PcrA helicase reveals a role in coupling ATP hydrolysis to strand separation. Nucleic Acids Res. 27, 3310–3317 10.1093/nar/27.16.3310 10454638 PMC148564

[17] Theis K., Chen P.J., Skorvaga M., Houten B.V. and Kisker C. (1999) Crystal structure of UvrB, a DNA helicase adapted for nucleotide excision repair. EMBO J. 18, 6899–6907 10.1093/emboj/18.24.6899 10601012 PMC1171753

[18] Kim J.L., Morgenstern K.A., Griffith J.P., Dwyer M.D., Thomson J.A., Murcko M.A. (1998) Hepatitis C virus NS3 RNA helicase domain with a bound oligonucleotide: the crystal structure provides insights into the mode of unwinding. Structure 6, 89–100 10.1016/S0969-2126(98)00010-0 9493270

[19] Caruthers J.M. and McKay D.B. (2002) Helicase structure and mechanism. Curr. Opin. Struct. Biol. 12, 123–133 10.1016/S0959-440X(02)00298-1 11839499

[20] Pause A., Méthot N. and Sonenberg N. (1993) The HRIGRXXR region of the DEAD box RNA helicase eukaryotic translation initiation factor 4A is required for RNA binding and ATP hydrolysis. Mol. Cell. Biol. 13, 6789–6798 10.1128/MCB.13.11.6789 8413273 PMC364741

[21] Caruthers J.M., Johnson E.R. and McKay D.B. (2000) Crystal structure of yeast initiation factor 4A, a DEAD-box RNA helicase. Proc. Natl. Acad. Sci. U.S.A. 97, 13080–13085 10.1073/pnas.97.24.13080 11087862 PMC27181

[22] Tai C.L., Pan W.C., Liaw S.H., Yang U.C., Hwang L.H. and Chen D.S. (2001) Structure-based mutational analysis of the hepatitis C virus NS3 helicase. J. Virol. 75, 8289–8297 10.1128/JVI.75.17.8289-8297.2001 11483774 PMC115073

[23] Richmond E. and Peterson C.L. (1996) Functional analysis of the DNA-stimulated ATPase domain of yeast SWI2/SNF2. Nucleic Acids Res. 24, 3685–3692 10.1093/nar/24.19.3685 8871545 PMC146154

[24] Nongkhlaw M., Dutta P., Hockensmith J.W., Komath S.S. and Muthuswami R. (2009) Elucidating the mechanism of DNA-dependent ATP hydrolysis mediated by DNA-dependent ATPase A, a member of the SWI2/SNF2 protein family. Nucleic Acids Res. 37, 3332–3341 10.1093/nar/gkp178 19324887 PMC2691824

[25] Nongkhlaw M., Gupta M., Komath S.S. and Muthuswami R. (2012) Motifs Q and I are required for ATP hydrolysis but not for ATP binding in SWI2/SNF2 proteins. Biochemistry 51, 3711–3722 10.1021/bi2014757 22510062

[26] Coleman M.A., Eisen J.A. and Mohrenweiser H.W. (2000) Cloning and characterization of HARP/SMARCAL1: a prokaryotic HepA-related SNF2 helicase protein from human and mouse. Genomics 65, 274–282 10.1006/geno.2000.6174 10857751

[27] Postow L., Woo E.M., Chait B.T. and Funabiki H. (2009) Identification of SMARCAL1 as a Component of the DNA Damage Response. J. Biol. Chem. 284, 35951–35961 10.1074/jbc.M109.048330 19841479 PMC2791023

[28] Yusufzai T., Kong X., Yokomori K. and Kadonaga J.T. (2009) The annealing helicase HARP is recruited to DNA repair sites via an interaction with RPA. Genes Dev. 23, 2400–2404 10.1101/gad.1831509 19793863 PMC2764493

[29] Boerkoel C.F., Takashima H., John J., Yan J., Stankiewicz P., Rosenbarker L. (2002) Mutant chromatin remodeling protein SMARCAL1 causes Schimke immuno-osseous dysplasia. Nat. Genet. 30, 215–220 10.1038/ng821 11799392

[30] Gupta M., Mazumder M., Dhatchinamoorthy K., Nongkhlaw M., Haokip D.T., Gourinath S. (2015) Ligand-induced conformation changes drive ATP hydrolysis and function in SMARCAL1. FEBS J. 282, 3841–3859 10.1111/febs.13382 26195148

[31] Sharma T., Bansal R., Haokip D.T., Goel I. and Muthuswami R. (2015) SMARCAL1 Negatively Regulates C-Myc Transcription By Altering The Conformation Of The Promoter Region. Sci. Rep. 5, 17910 10.1038/srep17910 26648259 PMC4673416

[32] Banroques J., Cordin O., Doere M., Linder P. and Tanner N.K. (2008) A conserved phenylalanine of motif IV in superfamily 2 helicases is required for cooperative, ATP-dependent binding of RNA substrates in DEAD-box proteins. Mol. Cell. Biol. 28, 3359–3371 10.1128/MCB.01555-07 18332124 PMC2423170

[33] Patne K., Rakesh R., Arya V., Chanana U.B., Sethy R., Swer P.B. (2017) BRG1 and SMARCAL1 transcriptionally co-regulate DROSHA, DGCR8 and DICER in response to doxorubicin-induced DNA damage. Biochim. Biophys. Acta 1860, 936–951 10.1016/j.bbagrm.2017.07.003 28716689

[34] , Sethy R., Rakesh R., Patne K., Arya V., Sharma T., Haokip D. (2018) Regulation of ATM and ATR by SMARCAL1 and BRG1, 10.1101/261610 30317028

[35] Story R.M., Li H. and Abelson J.N. (2001) Crystal structure of a DEAD box protein from the hyperthermophile Methanococcus jannaschii. Proc. Natl. Acad. Sci. U.S.A. 98, 1465–1470 10.1073/pnas.98.4.1465 11171974 PMC29280

[36] Graves-Woodward K.L., Gottlieb J., Challberg M.D. and Weller S.K. (1997) Biochemical analyses of mutations in the HSV-1 helicase-primase that alter ATP hydrolysis, DNA unwinding, and coupling between hydrolysis and unwinding. J. Biol. Chem. 272, 4623–4630 10.1074/jbc.272.7.4623 9020191

[37] Moolenaar G.F., Visse R., Ortiz-Buysse M., Goosen N. and van de Putte P. (1994) Helicase motifs V and VI of the Escherichia coli UvrB protein of the UvrABC endonuclease are essential for the formation of the preincision complex. J. Mol. Biol. 240, 294–307 10.1006/jmbi.1994.1447 8035457

[38] Yao N., Hesson T., Cable M., Hong Z., Kwong A.D., Le H.V. (1997) Structure of the hepatitis C virus RNA helicase domain. Nat. Struct. Biol. 4, 463–467 10.1038/nsb0697-463 9187654

[39] Subramanya H.S., Bird L.E., Brannigan J.A. and Wigley D.B. (1996) Crystal structure of a DExx box DNA helicase. Nature 384, 379–383 10.1038/384379a0 8934527

[40] Korolev S., Hsieh J., Gauss G.H., Lohman T.M. and Waksman G. (1997) Major domain swiveling revealed by the crystal structures of complexes of E. coli Rep helicase bound to single-stranded DNA and ADP. Cell 90, 635–647 10.1016/S0092-8674(00)80525-5 9288744

[41] Thompson J.D., Higgins D.G. and Gibson T.J. (1994) CLUSTAL W: improving the sensitivity of progressive multiple sequence alignment through sequence weighting, position-specific gap penalties and weight matrix choice. Nucleic Acids Res. 22, 4673–4680 10.1093/nar/22.22.4673 7984417 PMC308517

