# Supplementary material for: RecA-like domain 2 of DNA-dependent ATPase A domain, a SWI2/SNF2 protein, mediates conformational integrity and ATP hydrolysis
Source: Biosci Rep. 2018 Jun 27;38(3):BSR20180568. doi: 10.1042/BSR20180568 (PMC6019379; doi:10.1042/BSR20180568)
Supplement: Supplementary file 1 [file bsr20180568_Supp1.pdf]

Supplementary File

REC A-LIKE DOMAIN 2 OF ACTIVE DNA-DEPENDENT ATPASE A DOMAIN, A SWI2/SNF2 PROTEIN, MEDIATES CONFORMATIONAL INTEGRITY AND ATP HYDROLYSIS

Ritu Bansal, Vijendra Arya, Ramesh Sethy, Radhakrishnan Rakesh and Rohini Muthuswami
School of Life Sciences, Jawaharlal Nehru University, New Delhi 110067, India

SUPPLEMENTARY FIGURE LEGENDS

Supplementary Figure 1

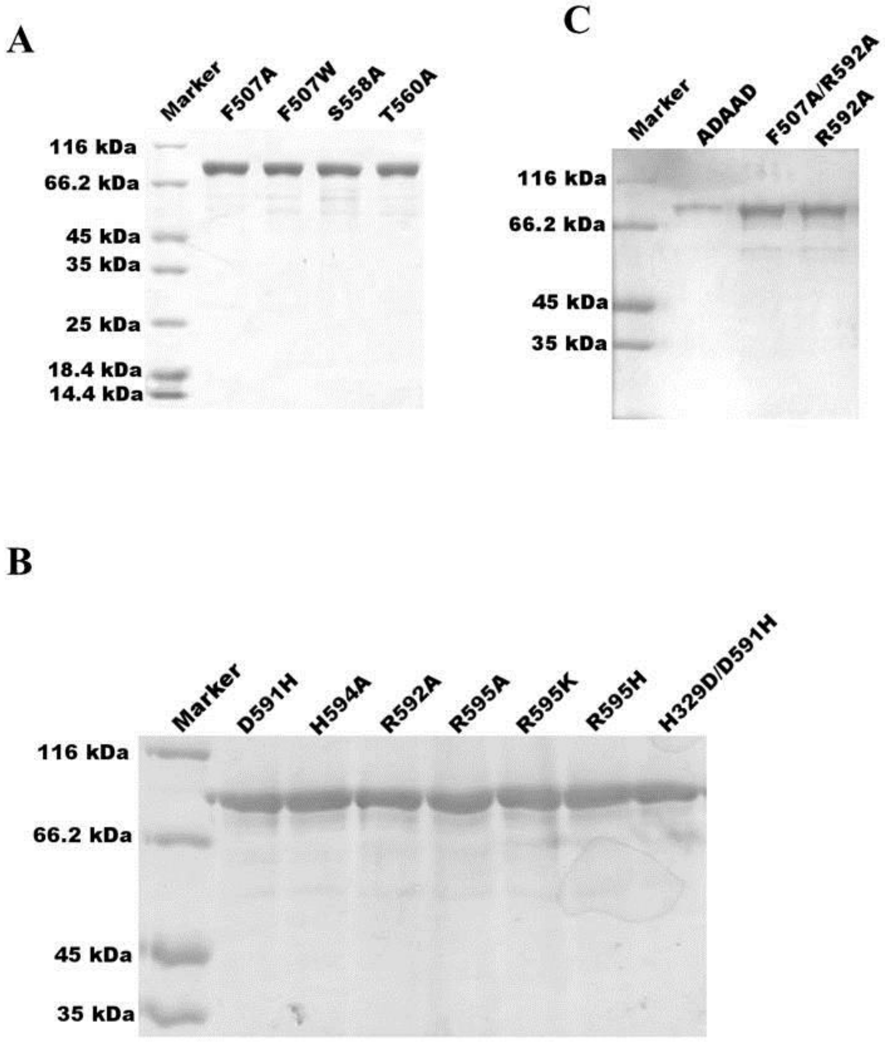

Supplementary Figure 1. Purification of the wild type and mutant proteins. (A). Motif IV mutant proteins- F507A and F507- and motif V mutant proteins-S558A and T560A. (B) Motif VI mutant proteins: D591H, H594A, R592A, R595A, R595K, R595H and H329D/D591H. (C) Wild type ADAAD, F507A/R592A and R592A.

Supplementary Figure 2

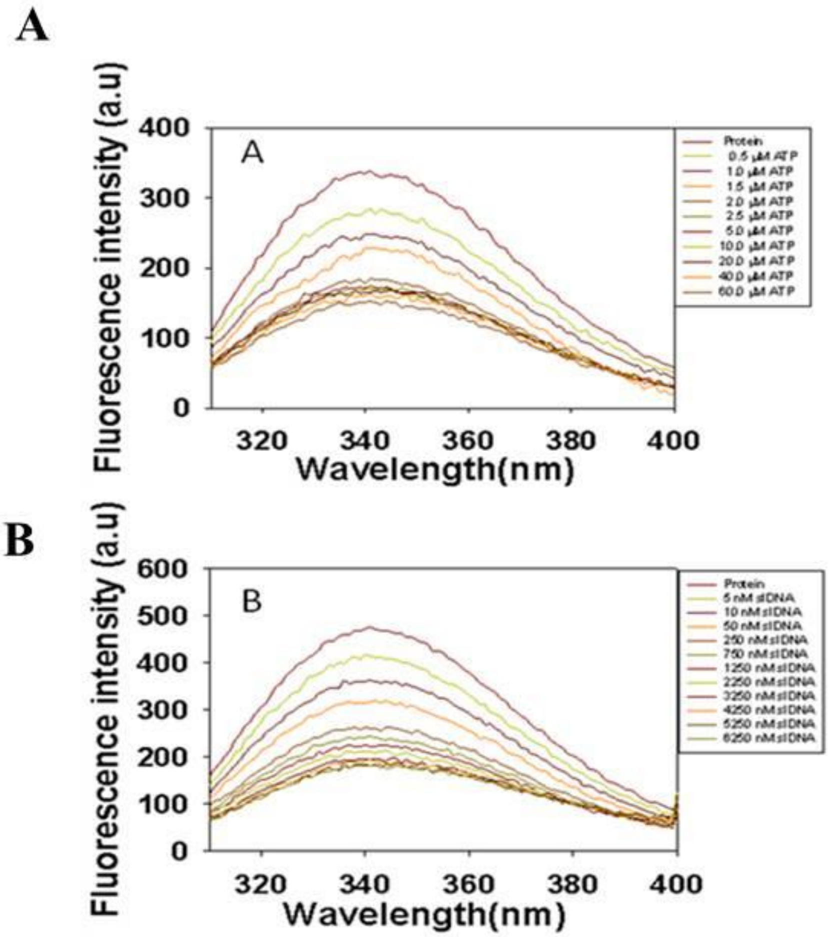

Supplementary Figure 2. Representative fluorescence spectra of ADAAD titrated with (A). ATP; (B) DNA.

Supplementary Figure 3

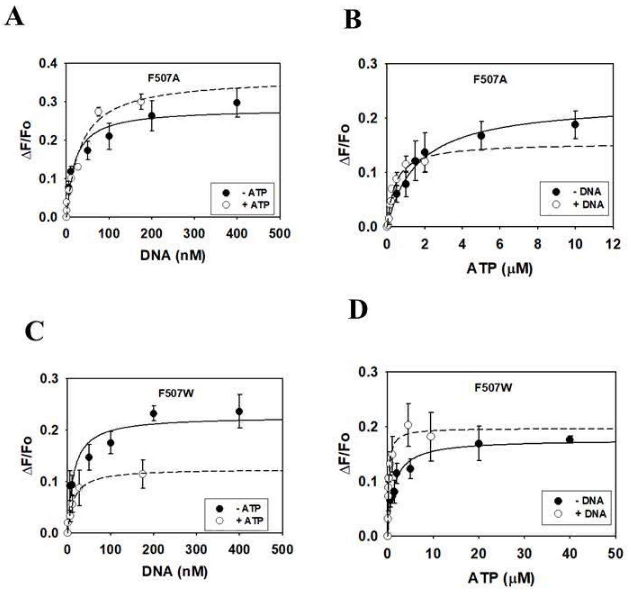

Supplementary Figure 3. Interaction of F507A and F507W with ligands was studied using fluorescence spectroscopy. (A) Interaction of F507A with DNA in the absence and presence of saturating concentration of ATP. (B) Interaction of F507A with ATP in the absence and presence of saturating concentration of DNA. (C) Interaction of F507W with DNA in the absence and presence of saturating concentration of ATP. (D) F507W with ATP in the absence and presence of saturating concentration of DNA. All the titrations were done using fluorescence spectroscopy and represent the average ± standard deviation of two independent experiments. The protein concentration used in these experiments was 0.5 μM. was All the data were fitted using one-site saturation model.

Supplementary Figure 4

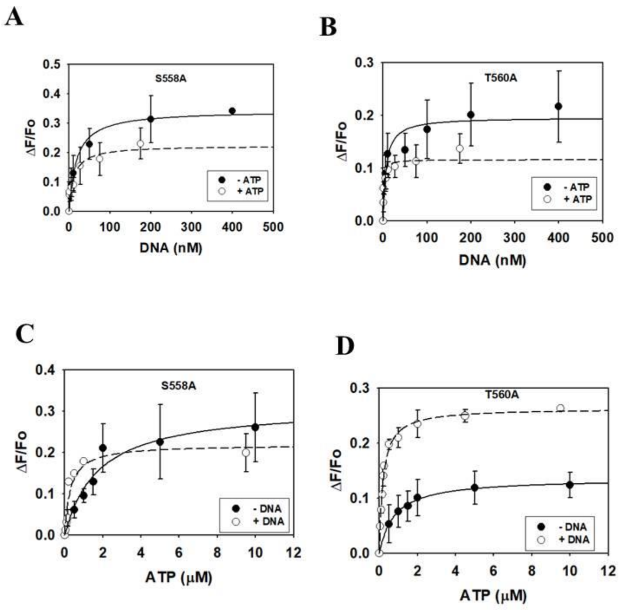

Supplementary Figure 4. Interaction of S558A and T560A with ligands was studied using fluorescence spectroscopy. (A) Interaction of S558A with DNA in the absence and presence of saturating concentration of ATP. (B) Interaction of T560A with DNA in the absence and presence of saturating concentration of ATP. (C) Interaction of S558A with ATP in the absence and presence of saturating concentration of DNA. (D) Interaction of T560A with ATP in the absence and presence of saturating concentration of DNA. All the titrations were done using fluorescence spectroscopy and represent the average ± standard deviation of two independent experiments. The protein concentration used in these experiments was 0.5 μM. All the data were fitted using one-site saturation model.

Supplementary Figure 5

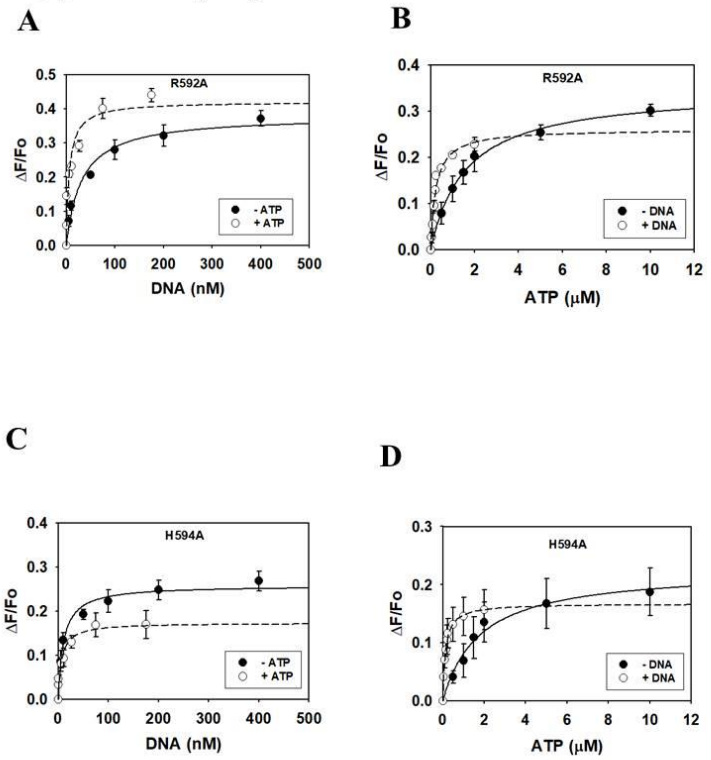

Supplementary Figure 5. Interaction of R592A and H594A with the ligands was studied using fluorescence spectroscopy. (A) Interaction of R592A with DNA in the absence and presence of saturating concentration of ATP. (B) Interaction of R592A with ATP in the absence and presence of saturating concentration of DNA. (C) Interaction of H594A with DNA in the absence and presence of saturating concentration of ATP. (D) Interaction of H594A with ATP in the absence and presence of saturating concentration of DNA. All the titrations were done using fluorescence spectroscopy and represent the average ± standard deviation of two independent experiments. The protein concentration used in these experiments was 0.5 μM. All the data were fitted using one-site saturation model.

Supplementary Figure 6

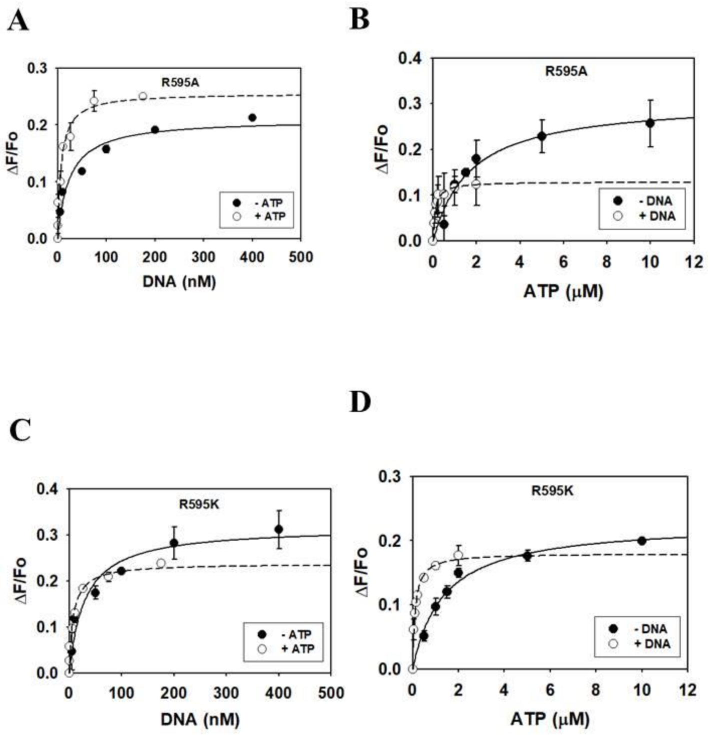

Supplementary Figure 6. Binding affinities were calculated of R595A and R595K for interaction with ATP and DNA using fluorescence spectroscopy. (A) Interaction of R595A with DNA in the absence and presence of saturating concentration of ATP. (B) Interaction of R595A with ATP in the absence and presence of saturating concentration of DNA. (C) Interaction of R595K with DNA in the absence and presence of saturating concentration of ATP. (D) Interaction of R595K with ATP in the absence and presence of saturating concentration of DNA. All the titrations were done using fluorescence spectroscopy and represent the average ± standard deviation of two independent experiments. The protein concentration used in these experiments was 0.5 μM. All the data were fitted using one-site saturation model.

Supplementary Figure 7

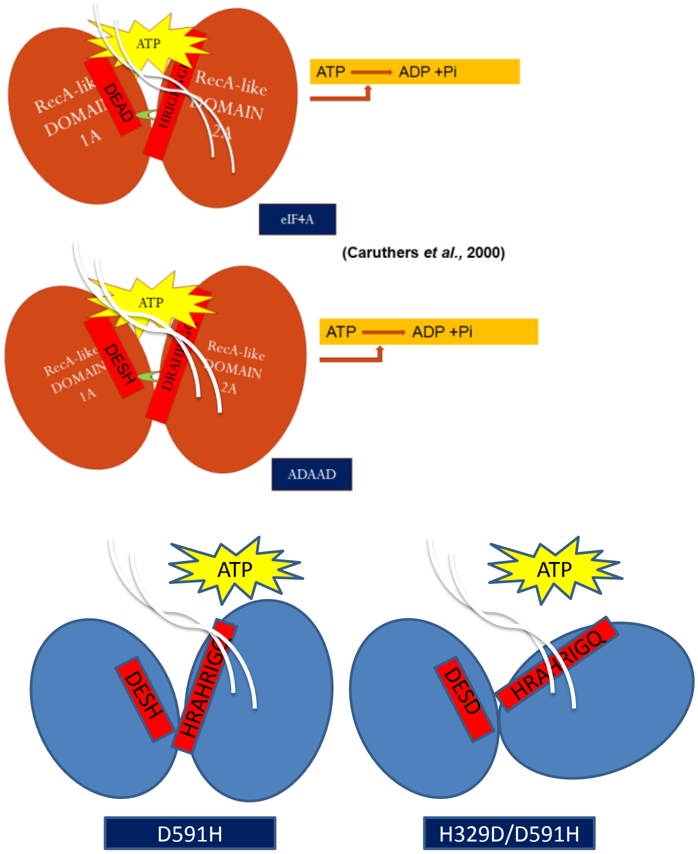

Supplementary Figure 7. (A) Diagrammatic representation depicting inter-lobe communication in eIF4 [1] (B) In ADAAD the covariance in the positions of aspartate and histidine is observed leading us to ask whether a possible salt bridge exists between motif II and motif VI. (C) To test the hypothesis, the aspartate (D591) present in motif VI was mutated to histidine which resulted in abrogation of ATPase activity. (D) The ATPase activity was not restored when the histidine (H329) present in motif II was mutated to aspartate.

Supplementary Figure 8

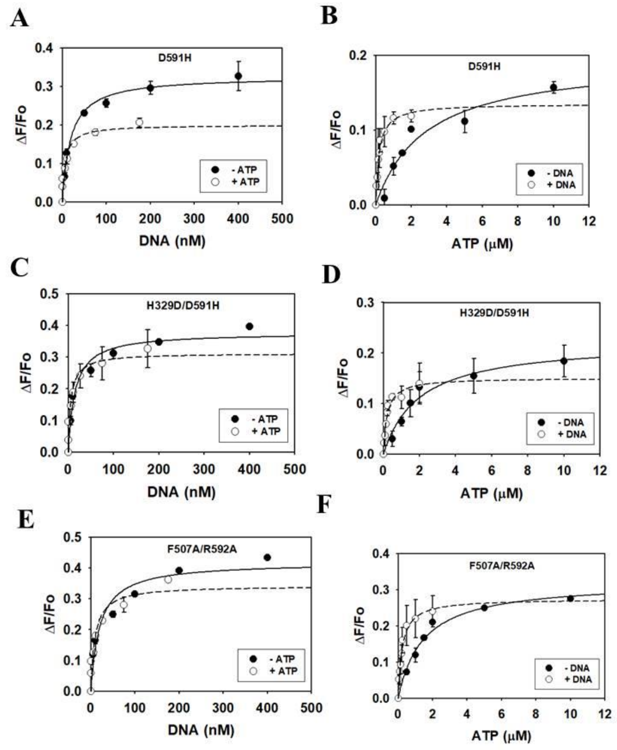

Supplementary Figure 8. The inter-lobe and intra-lobe interactions were studied using fluorescence spectroscopy. (A) Interaction of D591H with DNA in the absence and presence of saturating concentration of ATP. (B) Interaction of D591H with ATP in the absence and presence of saturating concentration of DNA. (C) Interaction of H329D/D591H with DNA in the absence and presence of saturating concentration of ATP. (D) Interaction of H329D/D591H with ATP in the absence and presence of saturating concentration of DNA. (E) Interaction of F507A/R592A with DNA in the absence and presence of saturating concentration of ATP. (F) Interaction of F507A/R592A with ATP in the absence and presence of saturating concentration of DNA. All the titrations were done using fluorescence spectroscopy and represent the average ± standard deviation of two independent experiments. The protein concentration used in these experiments was 0.5 μM. All the data were fitted using one-site saturation model.

Supplementary Figure 9

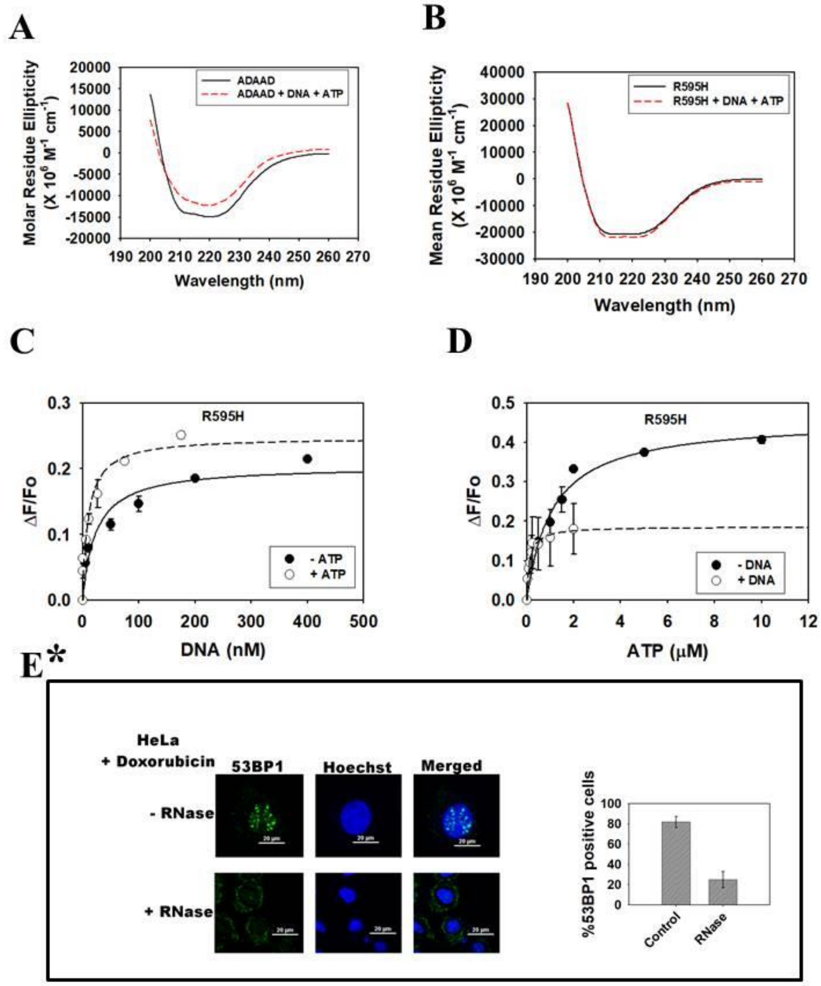

Supplementary Figure 9. The DNA damage response pathway is impaired in the SIOD-associated mutant R820H. (A). Comparison of the CD spectra of wild type ADAAD in the absence and presence of ligands. (B). Comparison of the CD spectra of R595H in the absence and presence of ligands. (C). Interaction of R595H with DNA in the absence and presence of saturating concentration of ATP. (D). Interaction of R595H with ATP in the absence and presence of saturating concentration of DNA. All the titrations were done using fluorescence spectroscopy and represent the average ± standard deviation of two independent experiments.

The protein concentration used in these experiments was 0.5 μM. All the data were fitted using one-site saturation model. (E) Formation of 53BP1 was monitored in HeLa cells after treatment with 2 μM doxorubicin for 10 min in the absence and presence of RNase. This data has been reported in [2] and is presented here to show the alteration in number of 53BP1 positive cells when HeLa cells are treated with doxorubicin and RNase.

REFERENCES

1 Caruthers, J. M., Johnson, E. R. and McKay, D. B. (2000) Crystal structure of yeast initiation factor 4A, a DEAD-box RNA helicase. Proc. Natl. Acad. Sci. **97**, 13080–13085.

2 Sethy, R., Rakesh, R., Patne, K., Arya, V., Sharma, T., Haokip, D., Kumari, R. and Muthuswami, R. (2018) Regulation of ATM and ATR by SMARCAL1 and BRG1.

Supplementary Table 1: List of primers used for making site-directed mutants

**Mutant**	**Forward primer (5′ 3′)**	**Reverse primer (5′ 3′)**	**T_m ^oC**
F507A	GTTTCTTGTGGCTGCGCACCATAAG	CTTATGGTGCGCAGCCACAAGAAAC	60
F507W	GTTTCTTGTGTGGGCGCACCATAAG	CTTATGGTGCGCCCACACAAGAAAC	55
S558A	GCGTGCTGGCTATCACCGCC	GGCGGTGATAGCCAGCACGC	60
T560A	GCTGTCCATCGCTGCCGCCAACATG	CATGTTGGCGGCAGCGATGGACAGC	60
D591H	GCTGAGCATCGGGTGCACCGCATC	GATGCGGTGCACCCGATGCTCAGC	50
R592A	GCTGAGGACGCGGTGCACCGCATC	GATGCGGTGCACCGCGTCCTCAGC	50
H594A	GACCGGGTGGCACGCATCGGACAA	TTGTCCGATGCGTGCCACCCGGT	50
R595K	GACCGGGTGCACAAGATCGGACAA	TTGTCCGATCTTGTGCACCCGGTC	50
R595A	GACCGGGTGCACGCCATCGGACAA	TTGTCCGATGGCGTGCACCCGGTC	50
R595H	CACCATATCGGACAATTGAG	CTCAATTGTCCGATATGGTG	
R820H	CGCGTGCACCATATTGGACAGACCAGCTCC	GGAGCTGGTCTGTCCAATATGGTGCACGCG	

Supplementary Table 2: Primers used for qPCR.

Genes	Forward primer (5′→3′)	Reverse primer (5′→3′)
SMARCAL1	CACCAAGGACAAAACTAAACAG	GTCCAAGATATATTCAATGACAGATG
BRG1	GCGAGTACGGCTGCAGGCT	TGGCGCAGCTGCCTCTG
DROSHA	GATCAGGTGGGAGATTCTACA	GGAACTCTCTAAATCTTCATCGAG
DGCR8	CGGGCGCCTCAGGTAGAAG	GCTGGCCACATTGCTCTTTTC
DICER	CGAGCCTCCATTGTTGGTCCAC	CCAGTTCGCCAATTTTGTGCAG
GAPDH	GGTCGGAGTCAACGGATT	GAGGGATCTCGCTCCTGG
